# Cell cycle correlated genes dictate the prognostic power of breast cancer gene lists

**DOI:** 10.1186/1755-8794-1-11

**Published:** 2008-04-25

**Authors:** Jonathan D Mosley, Ruth A Keri

**Affiliations:** 1 Department of Pharmacology, Case Western Reserve University School of Medicine, Cleveland, USA; 2 Division of General Medical Sciences – Oncology Case Western Reserve University School of Medicine, Cleveland, USA

## Abstract

**Background:**

Numerous gene lists or "classifiers" have been derived from global gene expression data that assign breast cancers to good and poor prognosis groups. A remarkable feature of these molecular signatures is that they have few genes in common, prompting speculation that they may use distinct genes to measure the same pathophysiological process(es), such as proliferation. However, this supposition has not been rigorously tested. If gene-based classifiers function by measuring a minimal number of cellular processes, we hypothesized that the informative genes for these processes could be identified and the data sets could be adjusted for the predictive contributions of those genes. Such adjustment would then attenuate the predictive function of any signature measuring that same process.

**Results:**

We tested this hypothesis directly using a novel iterative-subtractive approach. We evaluated five gene expression data sets that sample a broad range of breast cancer subtypes. In all data sets, the dominant cluster capable of predicting metastasis was heavily populated by genes that fluctuate in concert with the cell cycle. When six well-characterized classifiers were examined, all contained a higher than expected proportion of genes that correlate with this cluster. Furthermore, when the data sets were globally adjusted for the cell cycle cluster, each classifier lost its ability to assign tumors to appropriate high and low risk groups. In contrast, adjusting for other predictive gene clusters did not impact their performance.

**Conclusion:**

These data indicate that the discriminative ability of breast cancer classifiers is dependent upon genes that correlate with cell cycle progression.

## Background

Global gene expression profiling of breast tumors has provided considerable insight into the biological processes underlying this disease [1-3]. One application of gene expression data has been the development of prognostic "molecular signatures" or classifiers – short lists of genes that can be used to assign tumors to good or poor prognostic groups [4-11]. Some classifiers have now become commercially available diagnostic tools for breast cancer, making it imperative to understand how they function as well as how they differ from one another. A notable feature of published prognostic gene lists is that they have relatively little overlap in terms of composition. This lack of commonality has led to the proposition that prognostic lists use different genes to measure the same underlying biological process(es) [12,13]. Evidence supporting this notion includes a study evaluating the predictive abilities of five prognostic gene lists using a common gene expression data set, which showed that four of the five classifiers examined performed comparably in predicting future metastases despite having almost no overlap in constituent genes [14]. Where there is overlap between gene lists, the established function of many of the common genes is regulation of cell cycle progression [7]. Thus, gene expression signatures predictive of tumor grade, which are highly enriched in cell cycle genes, tend to have the greatest overlap with other gene lists [15,16]. It has further been demonstrated that, for some lists, the performance of the full list can be recapitulated using only the cell cycle-related genes contained within that list [15,17]. This indicates that cell cycle genes are sufficient to create an effective classifier [18]. In contrast to the sufficiency of known cell cycle regulators to predict outcome, the extent to which established classifiers actually depend upon cell cycle-correlated genes has not been determined.

Gene expression data contains numerous clusters, or groups, of genes whose patterns of expression are highly correlated across tumors [19,20]. As a result, the expression of many different genes can be used as surrogate measures for a common pathological process. Thus, prognostic gene lists need only to select a single gene from a highly correlated gene cluster to capture the overall expression pattern of all genes within that cluster. If each prognostic gene list utilized a different gene from a correlated set, the lists would have no overlap in gene identity, but would perform comparably because they would effectively measure the same cellular activity. We sought to determine whether this phenomenon was occurring with prognostic gene lists for breast cancer metastases. Herein, we describe the identification of clusters of correlated genes that are associated with the risk of developing metastases. We then determined the extent to which published classifiers utilized these gene clusters to predict outcome. Using five independent data sets, we show that all published prognostic gene lists that we examined rely upon a single group of correlated genes to predict outcome. Notably, the most predictive members of this cluster were genes which showed a cell-cycle pattern of regulation.

## Methods

### Previously published microarray datasets

Three publicly available breast cancer gene expression data sets were analyzed in the primary analyses: the Netherlands Clinical Institute (NKI2) data, which contains clinical (including metastatic recurrence latencies) and gene expression data on 295 women [9,21]; the Wang data set containing gene expression data on 296 women with lymph node negative disease [10] (GEO series GSE2034); and the KJX64 and KJ125 data sets containing data on 189 women, 64 of which were treated with tamoxifen, with primary operable invasive breast cancer [15] (GEO series GSE2990).

Gene expression data for the NKI2 data set were deposited as log10 expression ratios. For analyses identifying predictive clusters of correlated genes, a data set that contained no duplicated genes was created. Each probe in the data set was mapped to a gene symbol using the SOURCE database (source.stanford.edu). Gene symbols were identified for 21,220 probes. All probes that had complete data on more than 291 of the 295 subjects were used in the analysis. Missing values were imputed using the "impute" option from FastClus procedure (SAS). For genes for which there were multiple probes in the data set, the median expression value was used. The final data set contained complete data for 14,870 uniquely named genes. For all other analyses, all probes in the complete NKI2 data set were used.

For the Wang, KJX64 and KJ125 data sets, all probes (n = 22,286 in Wang and n = 22,285 in KJX64/KJ125) in each data set were used in all analyses. In the Wang data set, expression values were log2 transformed. Expression data from the KJX64 and KJ125 data sets was provided as log2 values. During data analyses, we noted that there were unexpectedly high rates of positive correlations among probes in the KJX64 and KJ125 data sets. To attenuate these correlations, expression data for each probe was normalized by dividing the values for each probe by the median expression value of all probes for a given subject.

### Simulated gene expression data set

As proof of concept of our iterative-subtractive approach for identifying the determinant groups of genes that dictate prognostication in a gene expression dataset, we began with a simulated gene expression data set. This data set was comprised of 300 subjects and contained 3 sets of correlated genes, two of which were associated with outcome, and was generated as follows. First, simulated expression data for 1000 uncorrelated genes was generated for each subject using a random number generator derived from a normal distribution (mean = 0, variance = 2). Next, we generated clusters of correlated genes: a "parent" gene was randomly selected from the 1000 genes and the expression of additional genes were then computed to have expression values equaling the "parent" gene's expression value plus a random number drawn from a normal distribution of values having a mean of 0 and variances ranging from 1 to 7. The degree of correlation was determined by the magnitude of the variance. Three "parent" genes were selected to generate correlated clusters of either 120 (parent genes 1 and 2) or 240 (parent gene 3) genes. The larger cluster was generated to demonstrate that the size of the cluster does not impact the analysis. Finally, event and latency data were randomly assigned based on expression quartiles of gene1 and gene2, with increased expression of these genes being associated with an increased risk of an event. For each of these genes, the respective risk of an "event" across quartiles was randomly determined with probabilities of 0%, 5%, 20% and 30% and the latencies in the instance of an event were randomly generated to have mean values of 5, 4, 3 and 2 years. After generating the simulated data, the univariate Cox proportional hazard ratios for gene1, gene2 and gene3 were 1.4 (p < 0.0001), 1.2 (p = 0.0007) and 0.9 (p = 0.06), respectively.

### Published gene lists

The published prognostic gene lists analyzed here were selected because they all effectively stratify patients into high and low risk prognostic groups using data sets comprised of both estrogen receptor (ER) negative and ER positive tumors. Using these classifiers, we assigned tumors to high and low risk groups following the authors' published methods, except where otherwise noted. When assignment of tumors to high and low risk groups involved the use of training sets, subjects used in the training sets were not included in the subsequent analyses evaluating the performance of the predictor. For each data set, these training sets were comprised of a random set of subjects, except for the NKI2 data set, where the training set for "good" prognosis subjects (subjects who did not experience metastases within 5 years) were the 30 subjects who did not experience metastases and where the conservFlag variable was either +1 or -1 [9]. The numbers of subjects used in training sets varied, hence the statistical analyses for the selected gene lists may involve different numbers of subjects and the relative performance of a list with respect to other lists may not be directly comparable. Gene symbols, accession numbers or probe identifiers from prognostic lists were extracted from materials accompanying the original publications and mapped to probe identifiers in each expression data set using Unigene cluster IDs derived from the SOURCE database. Unless otherwise noted, each instance of a matching probe was used in the analyses. For some prognostic gene lists, there were genes that could not be mapped to a probe in a given expression data set. A list of the number of probes used to compute prognostic scores and the numbers of subjects used in training sets is summarized [see Additional file 2].

The "70-gene" prognostic score was computed as described previously [9,11]. A 90% sensitivity (10% false positive) threshold was used to assign tumors to high and low risk groups. The "76-gene" predictor was computed as described by the authors, with one exception: a 90% sensitivity threshold was used to establish cut-off values for high and low risk tumors in the training sets rather than a 100% threshold since some tumors had outlying values in some data sets and this slightly lower threshold enhanced the performance of the classifier [10]. Weighted hazard ratio coefficients for all analyses were computed using unadjusted data. Because the "76-gene" predictor uses separate gene lists for ER positive and negative tumors, each list of genes was analyzed separately. The "Wound Signature" prognosticator was only examined in the NKI2 data sets since computations for this prognosticator relied upon centroid values previously computed by the authors [4]. The centroid values for "activated" (poor prognosis) tumors were obtained from the author's supplemental data files [4]. A 90% sensitivity classifier was used to define high and low risk groups. The "Histological grade signature" was computed as described by the authors, with the exception that a 90% sensitivity threshold was used for group assignment [15]. The probe identifiers for each of the data sets analyzed were previously mapped by the authors and were available as a supplement to the original publications. The Naderi prognostic score was computed as described by the authors, except that all probes mapped to each data set were individually used in the computations [6]. Finally, the 21 gene "Recurrence Score" was analyzed only in ER positive tumors. The score was computed as described by the authors [8]. In gene expression data sets where there were multiple probes for a given gene, the median value of the probes was used. Expression data for each gene analyzed was adjusted to have a mean value of 7.5 to ensure that expression values fell within the ranges reported by the authors. Since data adjustment precluded the use of fixed cut-off points to define high and low risk groups, as described by the authors, the performance of the predictor was analyzed by either ROC analysis or a 90% sensitivity classifier. Expression data from the NKI2 data were transformed to log2 values when computing the Recurrence score.

### Survival analyses

All survival analyses are based on 5-year metastatic recurrence latencies. All subjects not experiencing metastases within 5 years were censored at that time point. Cox proportional hazards regression models were used to measure the strength of the association between the covariates and recurrence (PHReg procedure). For most analyses, univariate proportional hazards ratios comparing good and poor prognostic groups are reported. For multivariable models, the most parsimonious set of covariates was determined using forward and backward selection methods on all clinical covariates contained within each dataset. In the NKI2 data set, these were determined to be age (in years), tumor size, ER status (ER positive vs. ER negative) and grade (1 vs. 2 and 1 vs. 3). In the KJX64/KJ125 data set, the covariates were tumor size (> 2 cm vs. < 2 cm) and grade (grade 2 vs. grade 1/3). ER status was the only clinical covariate contained in the Wang data set. Principal component variables were standardized to have a mean of 0 and standard deviation of 1 in proportional hazards models.

### Identifying predictive clusters of correlated genes

The following approach was taken to iteratively identify clusters of positively correlated genes associated with the risk of developing metastases in each data set. This method assigns a rank to each gene probe within a data set such that a highly ranked probe has the following properties: 1) it is strongly predictive of outcome (i.e. it has a low univariate hazard ratio (HR) p-value); 2) its expression is highly positively correlated with other probes; and 3) the HR p-values of the positively correlated probes decreases in direct proportion to their correlation with the gene being ranked. The rationale for the third criteria is that many probes may be associated with outcome solely because their expression pattern follows, to various degrees, the expression pattern for a highly predictive gene.

First, a univariate Cox proportional hazards p-value was computed for each probe to identify probes predictive of metastatic tumor latencies (PHReg procedure). Only probes with a HR p-value less than 0.05 were further analyzed in the breast cancer gene expression data sets. For the simulated data set, all probes with p-values less than 0.5 were analyzed. Next, for each probe, its linear Pearson's correlation coefficient (r) to every other probe was calculated. A new Pearson's correlation coefficient (corr) and covariance (cov) value was then computed for each probe using the correlation coefficients (r) and the log10(HR p-values); these new correlation and covariance values were computed using only those genes that were positively correlated with the probe being examined. Each probe was then assigned a score based on the following computation:

Score = AbsoluteValue(Cov (r, log10(HR p-value))) * Corr(r, log10(HR p-value)).

A graphical representation of two potential outcomes of the scoring procedure is provided [see Additional file 1]. The covariance component of this score gives increased weight to probes that are positively correlated with a large number of genes that are highly predictive of survival (i.e. have small p-values). The Pearson's correlation component of this score gives increased weight to those probes for which there is a linear relationship between its correlations with all other probes and their HR p-values. In total, this score ranks each probe in the data set such that those with the lowest (*i.e*. most negative) values are the most predictive members of clusters of correlated genes associated with the risk of developing metastases.

After assigning scores to each probe, the top 10 ranked genes (the genes with the lowest scores) were selected as representatives of the cluster. If the expression levels for these genes was highly correlated across tumors, this would indicate that a cluster of correlated genes associated with metastases had been identified. Having identified a cluster, the "average" expression pattern of the 10 genes was measured by computing the first principal component (PC) for the 10 genes (Princomp procedure). The PC represents a new variable that is maximally correlated with the expression values of the top 10 genes. We then used the PC variable to globally adjust the data set thereby eliminating correlations between the top 10 genes and all other genes (described below). After this global adjustment, the next predictive cluster of genes could be identified by repeating this entire scoring process using the adjusted data.

### Global adjustment of gene expression data

Identification of subsidiary principal components required the selective elimination of correlations between probes and a previously identified PC variable. To accomplish this, the expression data were globally adjusted for either an individual gene probe or a principal component (PC) variable by fitting a least-squares regression line (which included an intercept term) to each probe in the dataset and then computing the residuals (GLM procedure). Each probe was the dependent variable and the probe or the PC being adjusted for was the continuous independent variable. The residuals (adjusted values) for each probe represent the new expression values for that probe. This adjustment removes the variance from each probe that is linearly explained by the independent variable. The consequence of adjustment is that the adjusted value (residual) for a probe is no longer linearly correlated with the independent variable. Thus, probes that are predictive of metastases solely because their expression levels are correlated with the independent variable will no longer be predictive after adjustment. As a control for the effects of globally adjusting data, expression values were adjusted using a regression model that contained an intercept term only, but no other covariates.

### Statistical analyses

All calculations were performed using SAS version 9.1 (SAS Institute, Cary, NC). All statistical tests were two-sided. All correlation values represent Pearson's correlation coefficients. Receive operator characteristic (ROC) curves were generated using the Logistic procedure and were based on 5 year metastatic recurrence rates.

In the NKI2 data, expression values for probes typically fell between the ranges of -1 and +1. In some instances, the values for some probes represented extreme outliers. Thus, observations for probes with absolute values greater than 1.9 were omitted from these analyses.

## Results

### Development of a method for iteratively identifying clusters of correlated genes associated with an outcome

The multiple prognostic gene lists for breast cancer that have been described have little overlap in their gene composition. We developed a method to sequentially identify clusters of correlated genes that control the prognostic power of these gene lists with the goal of identifying the common underlying biological features of these classifiers. Specifically, we devised an approach to identify clusters of genes that are independently predictive of metastases.

**Figure 1:**
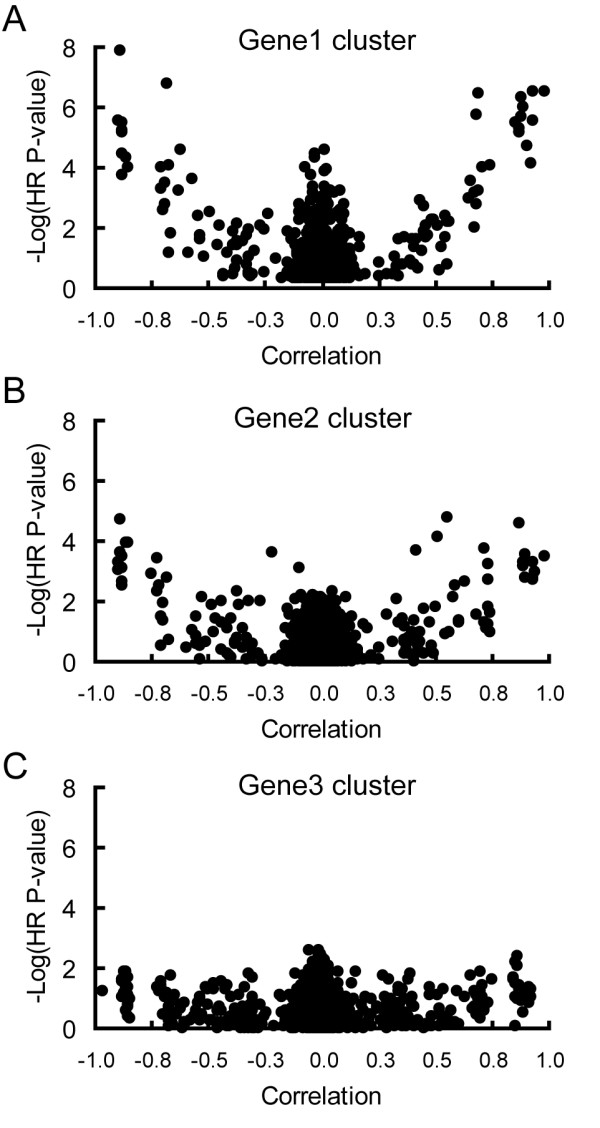
**Identification of predictive, correlated gene clusters in a simulated data set**. A simulated expression data set that included 3 independent correlated gene clusters, two of which contained a gene associated with outcome (gene1 and gene2), as well as an additional set of uncorrelated probes was generated as described in the Methods. Each figure shows data for 901 simulated genes with a univariate hazard ratio (HR) p-value less than 0.5. Each graph is a scatter plot of the negative log of the p-value of the univariate HR for a gene versus its correlation to a principal component (PC) variable. The PC variable was derived from the expression values of the top 5 ranked genes representing the most predictive correlated gene cluster identified in the current iteration. A large value on the y-axis corresponds to a small p-value, indicating that a gene is strongly associated with outcome. A. The first correlated set of predictive genes identified on an analysis of unadjusted expression data. B. The second set of correlated genes identified after the expression data were adjusted for the first PC identified in graph (A). HR p-values were computed using the adjusted data. C. A third set of correlated genes was revealed after the data were sequentially adjusted for the PC variables identified in (A) and (B). Note that no additional correlated clusters of genes were identified that had small p-values, indicating that the 2 PC variables represented the two major clusters of genes predictive of outcome in the simulated data set, thereby confirming the efficacy of this approach.

As a proof of principle of this method, we simulated a gene expression data set that included 3 independent groups, or clusters, of correlated genes. In two of the three groups, we stipulated that one gene (named gene1 and gene2) was correlated with an outcome. In the third group, no genes were correlated with outcome. Using an algorithm designed to identify genes belonging to predictive clusters, we found that the top 10 ranked genes identified by this approach were gene1 and nine additional genes that were highly correlated with gene1. Using a principal component (PC) variable to represent the average expression of the top 5 ranked genes, a "cluster" of positively and negatively correlated genes predictive of outcome is easily visualized when plotting the p-value of each gene's hazard ratio (HR) against its correlation to this PC (Figure 1A). This cluster forms the boundaries of the "V-shaped" pattern seen on the graph. This pattern arises because the strength of an individual gene's relationship to the outcome is dictated by the extent to which its expression correlates with that of the PC. Genes with significant p-values that are not correlated to the PC represent additional genes that are predictive of outcome but are independent of this first gene cluster; these genes form the top of the cone in the center of the graph.

We then used the PC variable to globally adjust the gene expression data. This permitted repetition of the scoring process, leading to identification of a second correlated gene group that is independently predictive of outcome. In the next iteration, the top 10 ranked genes were gene2 and genes highly correlated with it. This cluster is shown in Figure 1B. Note that the overall magnitude of the p-values is smaller for this cluster since gene2 was less strongly associated with outcome than gene1. On the next iteration, a third correlated cluster of genes was identified that was not strongly associated with outcome (Figure 1C). In sum, these analyses demonstrate that clusters of correlated genes independently predictive of outcome can be sequentially identified, and that the order in which clusters are elucidated reflects the strength of their association with the outcome.

### Identification of correlated gene clusters associated with breast cancer metastases

**Figure 2:**
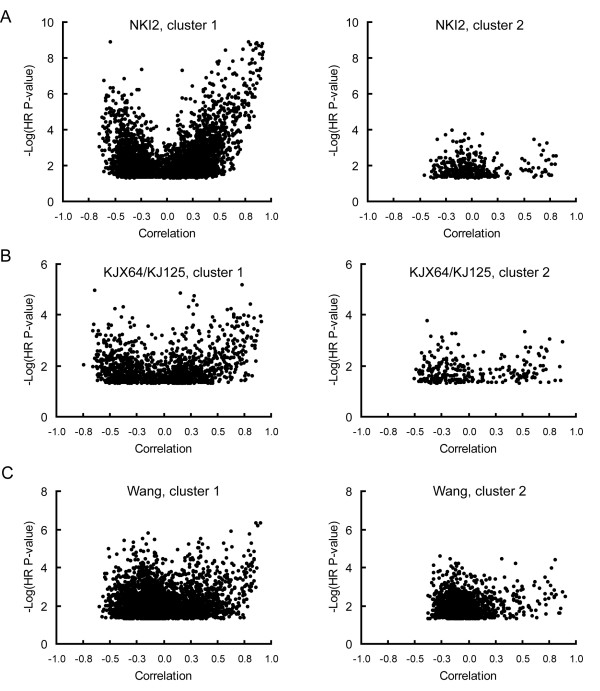
**Scatter plots showing the two most predictive clusters of correlated probes in three independent breast cancer gene expression data sets**. Each graph is a scatter plot of the negative log of the p-value for a univariate HR versus the correlation of each probe to the first PC variable derived from the expression values of the top 10 ranked probes. For each data set, only probes which had a univariate HR p-value of less than 0.05 for 5 year metastatic recurrence latencies were examined. A. Scatter plots for the 3,311 probes in the NKI2 dataset based on (left panel) unadjusted and (right panel) data adjusted for the first PC variable. B. Scatter plots for the 1,282 probes in the combined KJX64 and KJ125 datasets based on unadjusted (left panel) and data adjusted (right panel) for the first PC variable. C. Scatter plots for the 4,088 probes in the Wang dataset based on unadjusted (left panel) and data adjusted (right panel) for the first PC variable.

To determine the basis for a lack of commonality between breast cancer prognostic gene lists, we used the iterative-subtractive method described above to identify gene clusters associated with breast cancer metastases. We first evaluated the NKI2 data set, which is comprised of 295 women with early stage breast cancer [9]. In this data set, 3,311 genes had a univariate HR p-value for metastatic recurrence of less than 0.05. These genes were then used to identify groups of correlated genes that were predictive of metastatic progression. In the first iteration, the majority of the top-ranked genes in the first cluster were involved in cell-cycle regulation and function [see Additional file 2]. A graph of the correlations between all genes and a PC variable computed from the top 10 ranked genes in this cluster is shown in Figure 2A. A striking feature of this graph is that the vast majority of genes predictive of metastases are correlated with the cell-cycle PC. Only 380 of the 3,311 genes analyzed remained significantly associated with metastases (p < 0.05) after the expression data for each gene was adjusted for the PC. On the second iteration, the top-ranked genes were predominantly involved in protein translation [see Additional file 2]. Of note, only a few genes were strongly correlated with this PC, and their HR p-values were much lower than the genes highly correlated with the first PC (Figure 2A). Repeated iterations through this data set failed to identify any additional large clusters of correlated genes that were strongly associated with the risk of developing metastases [see Additional file 2].

This approach was repeated with the KJX64/KJ125 data sets, which are comprised of data from 189 women with primary operable invasive breast cancer [15]. Similar to the NKI2 data set, the top ranked predictive cluster of probes was dominated by cell cycle genes (Figure 2B) [see Additional file 2]. Of 1,282 probes initially analyzed, 255 retained significant HR p-values (p < 0.05) after adjusting for this PC. The top ranked probes in the second cluster were predominantly related to immune function (Figure 2B) [see Additional file 2]. Only 58 genes were still associated with outcome after adjusting the data for this second gene cluster. Additional predictive gene clusters were not found among these remaining genes.

Using a third, independent data set, we found that the primary cluster in the Wang data set of 296 women with lymph node negative disease [10] was also dominated by a set of cell cycle related genes (Figure 2C) [see Additional file 2]. Of the 4,088 probes initially analyzed, 1,490 remained predictive after adjusting for this cluster. The next cluster consisted of relatively few correlated genes predictive of outcome (Figure 2C) [see Additional file 2], and was comprised of genes potentially related to TGF-β or activin signaling [22]. This cluster was relatively small, and 850 genes were still predictive after adjustment. The top ranked genes in the third iteration were not consistently correlated with each other and, thus, did not define a correlated gene cluster [see Additional file 2].

In summary, the dominant predictive network in all three independent expression data sets was a cluster of correlated genes whose most predictive members were cell cycle-associated. Similar results were obtained when analyses were performed on two additional independent data sets [see Additional file 2], *i.e*. the primary PC was dominated by cell cycle correlated genes.

### Each principal component variable is independently predictive of metastases in multivariable analyses

Multivariable Cox proportional hazards regression analysis was performed to determine the relationship between each principal component (PC) variable generated in the previous analysis and the risk of metastasis. In each data set, the first PC variable (representing the cell cycle gene cluster) was significantly associated with metastases in all tumors as well as in the subset of ER positive tumors, but not the subset of ER negative tumors (Table 1). In the NKI2 and Wang data sets, additional PC variables, such as the one representing the TGF-β-like cluster (PC2 from the Wang data set), were also significantly predictive of recurrence latencies in ER negative tumors. Hence, each of the primary PC variables identified with the iterative-subtractive approach was independently predictive of the risk of metastases in all breast tumors as well as in ER positive tumors. This suggested that each of these could potentially be used to generate novel classifiers. We tested this supposition with each of the five data sets. In each case, the cell-cycle PC variable performed comparably or better than, the other previously characterized predictive lists that we examined [see Additional file 2]. However, it should be noted that the use of the same data sets to evaluate the PC classifiers may introduce intrinsic bias that could enhance their performance.

**Table 1:** Multivariable Cox proportional hazards analysis for each principal component variable^1^.

		All tumors		ER positive tumors		ER negative tumors	
			
**Data set**	**Variable**	**Hazard Ratio**	**p-value**	**Hazard Ratio**	**p-value**	**Hazard Ratio**	**p-value**
**NKI2**							
	PC1	1.8	0.0001	2.8	< 0.0001	0.9	0.61
	PC2	0.6	< 0.0001	0.7	0.004	0.7	0.03
	PC3	1.2	0.01	2.1	0.0002	1.1	0.37
	Age	0.9	0.0009	0.9	0.01	0.9	0.02
	Tumor size^2^	1.8	0.01	2.2	0.009	2.4	0.04
	Grade 2^3^	2.0	0.13	1.1	0.87		
	Grade 3^3^	2.2	0.09	1.3	0.55		
	ER+	0.9	0.67				
**KJX64/KJ125**							
	PC1	1.5	0.004	2.2	< 0.0001	0.9	0.74
	PC2	0.6	0.002	0.6	0.002	0.5	0.12
	Grade 2^4^	2.7	0.002	3.0	0.005	1.5	0.54
	Tumor size^2^	3.2	0.002	4.4	0.002	1.8	0.42
**Wang**							
	PC1	1.8	< 0.0001	1.8	< 0.0001	1.7	0.06
	PC2	1.6	< 0.0001	1.7	0.0003	1.6	0.02
	PC3	2.0	< 0.0001	2.2	< 0.0001	1.5	0.05
	ER+	0.8	0.26				

1. Up to three principal component variables (PC1-PC3), each derived from the top ranked probes for a set of correlated genes predictive of metastatic recurrence latencies, were included in the models. Hazard ratios for these PCs represent the change in hazards per standard deviation increase in the variable. A hazard ratio greater than 0 indicates that increasing levels of genes positively correlated with the PC variable are associated with an increased risk of metastasis.

2. Hazard ratios for tumor size represent differences in tumor sizes greater than 2 cm versus tumors less than 2 cm.

3. Hazard ratios for grades are relative to grade 1 tumors. Grade variables were not included in the analysis of ER negative tumors due to the low numbers of tumors representing the various grades.

4. Hazard ratios are for grade 2 versus other grades.

### Genes correlated with the cell cycle principal component are overrepresented on published prognostic gene lists

**Figure 3:**
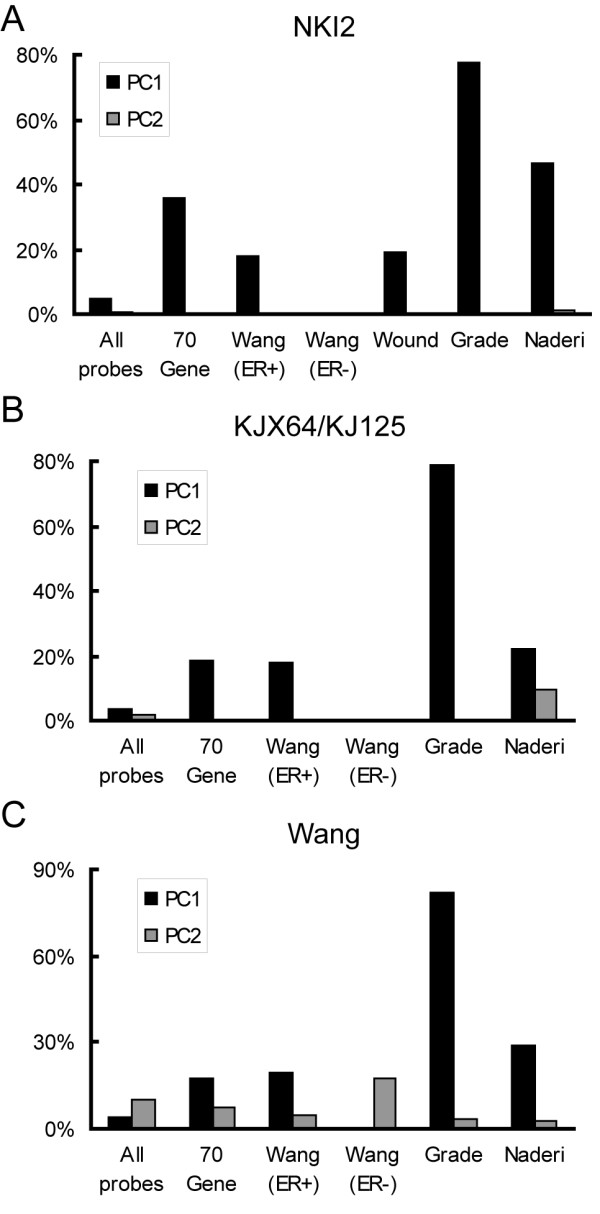
**Genes correlated with the cell cycle principal component are overrepresented on published gene lists**. A/B/C. Each bar graph shows the percentage of the genes within selected published prognostic gene lists that have Pearson's correlation coefficients greater than 0.4 or less than -0.4 to either the first (black bars) or second (grey bars) PC variables from each data set evaluated. The bars identified as "All probes" represent the percentage of all probes in the (A) NKI2 (n = 24,495), (B) KJX64/KJ125 (n = 22,285) and (C) Wang (n = 22,286) gene expression data sets that have correlations within the indicated ranges.

In all five data sets examined, the primary PC identified consisted of a large set of cell cycle-associated genes, suggesting that this gene cluster may significantly contribute to the predictive ability of various prognostic lists that were devised using such data. We determined the extent to which previously reported prognostic lists included genes from the correlated clusters by determining the percentage of genes on each list that were moderately to strongly correlated with the first two PC variables from each data set. Each prognostic gene list contained a higher than expected proportion of genes correlated with the cell cycle PC (Figure 3). For example, using the NKI2 expression data set, we found that only 1,200 of the 24,495 probes (*i.e*. 4.9%) had an absolute correlation with this PC greater than 0.4. In contrast, each of the previously reported classifiers we examined contained between 18% and 80% of probes with correlations of this magnitude. The one exception was the Wang predictor for ER negative tumors [10], which had no probes correlated with the cell cycle PC. The predictive list with the highest proportion of correlated probes was the grade signature, which was developed using ER positive tumors [15]. Likewise, when we examined other predictive gene lists derived from ER positive tumors, we found a similar over- representation of correlated genes. For example, in the proliferation signature [23], 100% of 50 probes were correlated to the cell cycle PC, while the consensus signature [7] had 59% of 66 probes with similar correlations when evaluated in the NKI2 data set (data not shown).

Few, if any, probes in the expression data sets were correlated with the secondary PC variables. The notable exception was the Naderi prognostic gene list [6] in the KJX64/KJ125 data set. Almost 10% (2–3 fold higher than expected) of probes within this predictor had absolute correlations greater than 0.4. In sum, these data show that most prognostic gene lists are comprised of a higher than expected number of genes whose expression is correlated with the primary PC variable which measures fluctuations in the cell cycle.

### Prognostic gene lists depend upon the cell cycle principal component to identify good and poor prognosis tumors

To determine the extent to which the published gene-based classifiers are dependent upon genes that are correlated with the PC variables, we assessed the impact of globally adjusting the data using each PC variable on the discriminating ability of the predictive lists. Most of the gene lists were able to classify tumors into good and poor prognosis groups with significant differences in recurrence latencies when data were globally adjusted using a simple linear regression model that included only an intercept term (Table 2). This indicated that the process of global adjustment itself did not negate the predictive abilities of the lists. In contrast, all but one of the gene lists lost their ability to appropriately stratify tumors into significantly different high and low risk groups following adjustment for the cell-cycle PC variable (Table 2). The exception was the Wang ER negative predictor, which remained predictive in the data set from which it was derived. In contrast to the first PC, all gene lists remained predictive when adjusted for either the second or third PC variables (Table 2 and data not shown). Similar results were obtained when the various PC variables were used to adjust two additional data sets [see Additional file 2].

**Table 2:** Prognostic gene lists rely on genes correlated with the first (cell cycle) principal component variable^1^.

		**Intercept adjusted**		**PC1 adjusted**		**PC2 adjusted**	
			
**Data set**	**Gene list**	**Good/Poor^2^**	**HR (p-value)**	**Good/Poor^2^**	**HR (p-value)**	**Good/Poor^2^**	**HR (p-value)**
**NKI2**							
	70 Gene	91/174	**5.8 (< 0.0001)**	30/235	1.2 (0.67)	90/175	**5.7 (< 0.0001)**
	Wang 76-gene (ER+)	85/60	**4.5 (< 0.0001)**	44/101	0.9 (0.84)	89/56	**2.7 (0.005)**
	Wang 76-gene (ER-)	6/29	**0.9 (0.88)**	5/30	1.2 (0.78)	8/27	0.5 (0.25)
	Wound Signature	74/221	**4.1 (0.0002)**	27/268	1 (0.93)	73/22	**3.9 (0.0002)**
	Sotiriou Grade	92/203	**4.7 (< 0.0001)**	25/270	0.8 (0.8)	91/204	**4.7 (< 0.0001)**
	Naderi	131/104	**2.5 (0.0006)**	123/112	0.4 (0.003)^2^	128/107	**2.4 (0.0014)**
**KJX64/KJ125**							
	70 Gene	38/111	**2.8 (0.03)**	15/134	0.7 (0.4)	39/110	**2.9 (0.03)**
	Wang 76-gene (ER+)	53/36	1.6 (0.32)	35/54	1.2 (0.7)	61/28	1.8 (0.24)
	Wang 76-gene (ER-)^4^	14/5	n/a	16/3	n/a	12/7	n/a
	Sotiriou Grade	46/143	**2.9 (0.03)**	35/154	1.7 (0.24)	46/143	**2.7 (0.03)**
	Naderi	71/56	**3.9 (0.004)**	69/58	1.6 (0.24)	72/55	**3.9 (0.004)**
**Wang**							
	70 Gene	49/197	**2.6 (0.005)**	22/224	0.9 (0.41)	44/202	**2.3 (0.015)**
	Wang 76-gene (ER+)	45/84	**6.3 (0.0005)**	30/99	2 (0.11)	49/80	**5.6 (0.0003)**
	Wang 76-gene (ER-)	26/16	**11.7 (0.0001)**	29/13	**8.2 (0.0001)**	15/27	**4.9 (0.03)**
	Sotiriou Grade	57/229	**2.5 (0.007)**	21/265	0.7 (0.25)	49/237	**2 (< 0.0001)**
	Naderi	106/110	**2.4 (0.0008)**	105/111	0.9 (0.66)	105/111	**3.1 (< 0.0001)**

1. Hazard ratios (HR) represent the change in risk for tumors classified as having a poor prognosis versus tumors classified as having a good prognosis using a univariate proportional hazards analysis. Gene expression data were independently adjusted using simple linear regression analysis for either an intercept only, the principal component representing the first correlated gene cluster identified (PC1) or the second correlated gene cluster identified (PC2) in each data set. Univariate HRs that are significantly (p < 0.05) greater than 1 are shown in bold.

2. Values in the Good/Poor column indicate the number of tumors assigned to the good and poor prognosis groups, respectively. The total number of tumors per gene list will vary depending upon the number of tumors used in training sets.

3. The fact that this HR is significantly less than 1 indicates that the classifier did not appropriately classify tumors into appropriate good and poor prognosis groups.

4. There were too few events among good and poor prognosis groups to perform statistical analyses in the KJX64/KJ125 data set.

**Figure 4:**
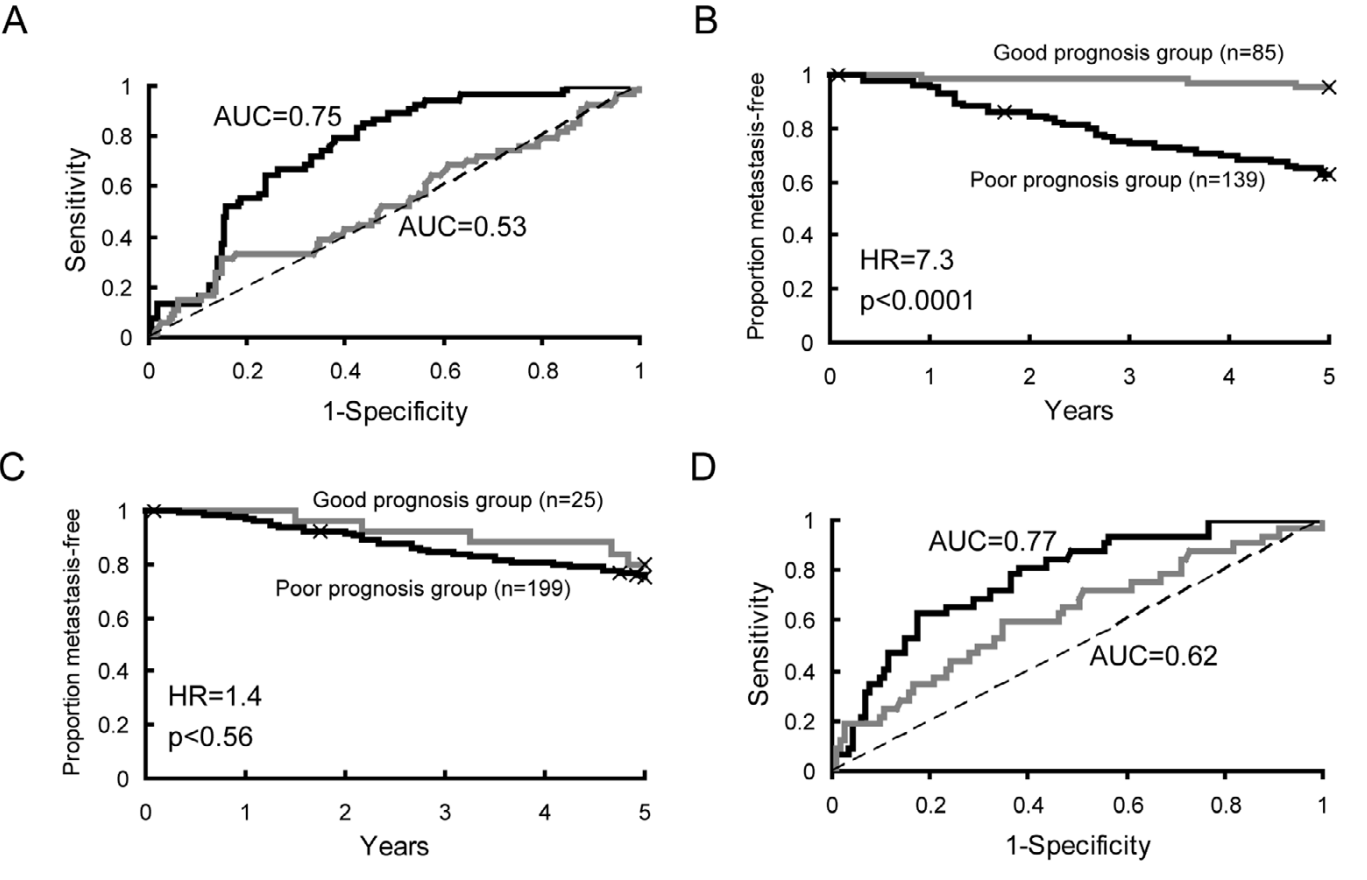
**The Recurrence Score prognosticator fails to perform appropriately after adjustment for the cell cycle principal component variable**. A. A ROC curve summarizing the performance of the 21-gene recurrence classifier in ER positive tumors in the NKI2 data set (n = 224 subjects) adjusted for either an intercept only (black line) or the cell cycle PC variable (grey line). The area under the curve (AUC) for each analysis is shown on the graph. The dotted line represents a ROC curve with an AUC of 0.5 (indicative of a low accuracy test). B/C. Kaplan-Meier plots for the recurrence score in the NKI2 data set for data adjusted for (B) intercept-only or (C) the cell cycle PC variable. Hazard ratios (HR) and p-values are from univariate proportional hazards analyses and represent the difference in hazards for poor versus good prognosis tumors. D. ROC curve for ER positive tumors in the KJX64/KJ125 data set (n = 147 subjects) adjusted for either an intercept only (black line) or the cell cycle PC variable (grey line).

We also examined the effect of adjusting for the PC variables on the 21-gene recurrence score predictor which functions as a clinically approved diagnostic tool for ER positive tumors [8]. In the NKI2 data set, the predictor robustly discriminated between high and low risk patients prior to adjustment (Figures 4A and 4B). However, after adjusting for the cell-cycle PC, the area-under-the-curve for the ROC plot decreased from 0.75 to 0.53 and the predictor was no longer capable of identifying appropriate high and low risk groups (Figures 4A and 4C). Similar results were obtained in the KJX64/KJ125 dataset, though the performance of the predictor was impacted to a lesser extent by adjustment (Figure 4D). Hazard ratios before and after adjustment were 6.9 (p = 0.002) and 2.2 (p = 0.18), respectively (90% sensitivity threshold). The recurrence score was not predictive in the Wang data set (HR = 2.4, p = 0.18), precluding an assessment of adjusting for the impact of the cell cycle PC on its function.

Collectively, these analyses revealed that prognostic gene lists rely on genes whose expression correlates with cell cycle genes to assign tumors to high and low risk groups across data sets. Further supporting this conclusion, we found that when data were adjusted for individual genes, rather than a PC variable representing a cluster of genes, the impact of such adjustment on the performance of the predictor was directly related to the magnitude of that gene's correlation with the cell cycle PC [see Additional file 3].

## Discussion

Herein, we described an approach to determine biological commonalities among non-overlapping gene-based classifiers that are predictive of breast cancer metastases. Although it has previously been suggested that cellular proliferation may be an underlying component of these classifiers, no studies have directly shown that this is, indeed, the case. To determine if there was a fundamental biological process that was consistently being interrogated by these prognostic lists, we developed a method to identify clusters of correlated genes that are associated with the risk of developing metastases in breast cancer. The advantage of this method is that it identifies large groups of correlated genes whose expression pattern is determined by common processes associated with an outcome. We suggest that such an approach is necessary when devising classifiers because all members of a correlated network function as a group to relay information regarding a common pathophysiological state [24]. More importantly, any one gene from a correlated cluster can act as a sentinel or reporter for all other genes within that same cluster. As a result, numerous prognostic lists can be generated which measure the same biological process but have little to no overlap in gene content. In addition, analytic approaches which examine individual probes are apt to identify only those correlated probes that are strongly associated with outcome, but not those probes with weaker correlations since they will only be modestly predictive [25].

Using an iterative-subtractive approach, we consistently show that the most predictive gene cluster in five independent, publicly available breast cancer data sets is represented by genes that either directly participate in the cell cycle or whose expression is dictated by cell cycle progression. Visualization of this cluster in the NKI2 gene expression data set was particularly striking as it revealed that the prognostic capacity of virtually every gene was directly related to that gene's correlation with cell cycle genes (Figure 2A). Indeed, when data were adjusted to selectively eliminate the correlations among the genes in this cluster, very few genes remained predictive of outcome. The highly correlated nature of all of the predictive genes in this data set explains why a comparison of the performance of five prognostic gene lists showed that 4 of 5 performed comparably even though these lists had very little overlap in their constituent genes [14]. It also reveals why using only the cell cycle correlated genes from a predictive gene list is sufficient to recapitulate the performance of the entire list [15,17,26] – because the other predictive genes in these lists function as weak proxies of the cell cycle genes.

A number of studies have identified cell cycle genes as being predictive of poor outcome within and across cancer types [23,27,28]. Cell cycle genes have also been shown to represent the molecular basis of tumor grade in breast cancer, a strong predictor of metastasis [15,16,29,30]. While cell cycle regulators may play a causative role in tumor development [31], their upregulation also likely represents an important unifying pathway for a number of tumor initiating events. As a result, the fundamental commonality among all breast cancer subtypes that predicts outcome is an increase in proliferation. This outcome further supports the notion that breast cancer is an amalgam of very different subtypes of disease, with each subtype having a distinct group of prognostic genes. When all of these classes of tumors are combined, the major common theme is cell cycle progression. This concept is further illustrated by the inherent lack of known mammary oncogenes, such as HER2/ErbB2 [32], in any of the predictive clusters we identified. A similar observation has been made in other studies which have used statistical methods to define prognostic gene sets [6,7,11]. While expression of cell cycle regulators is upregulated in HER2 tumors as well as ER negative tumors [19,33], HER2 and ER-α were either uncorrelated or modestly linearly correlated with the cell cycle clusters. The low correlations are likely due to the fact that these types of tumors represent a relatively small proportion of tumors in these data sets and that expression levels of these genes are uncorrelated with those for the cell cycle genes in the majority of tumor types. Thus, it is not surprising that these factors would not be the primary indicators of outcome, either by the method described herein or with other classifiers, when examining the entire set of tumors. In contrast, many of the genes negatively correlated with the cell cycle PC variable were genes that have previously been shown to define the good prognosis class of Luminal A tumors (e.g. SCUBE2, XBP1, FoxA1 etc.) [3,20] (data not shown). The negative correlations may arise either because these genes inhibit proliferation [34] or because they define a class of tumors with intrinsically low rates of proliferation. In either case, from the perspective of predicting metastasis latencies, they all function to various degrees as proxies for the cell cycle.

## Conclusion

Prognostic tools for breast cancer that utilize gene-based classifiers are currently being tested or used in clinical settings [35]. As more of these prognostic tools become commercially available, it will be imperative to have a thorough understanding of how they may differ in order to facilitate selection of the most appropriate test. Our studies suggest that these tools, despite having little overlap in gene content, rely heavily on a single metric to identify high and low risk tumors. The common biological process that is being measured is the upregulation of genes associated with cell proliferation. Consistent with this conclusion is the fact that the greatest overlap in established classifiers is typically genes encoding cell cycle regulators [7]. Furthermore, the cell cycle cluster of genes (PC1 for each data set) was as effective as all of the other established classifiers we examined when used to evaluate data sets other than those from which they were derived (Table 2) [see Additional file 2]. Most importantly, adjustment of the data sets for this cluster resulted in a loss of the predictive capacity for these classifiers, indicating that they require measuring fluctuations in the cell cycle correlated genes to effectively separate tumors into high and low risk groups. These findings suggest that validation of novel classifying gene lists should involve a comparison with a prognosticator comprised of a small number of cell cycle associated genes, as such a classifier may be able to capture the same amount of critical prognostic information with a smaller number of genes This study also underscores the fact that many genes are correlated with the cell cycle genes even though they may not be directly associated with this process. Thus, simply removing known cell cycle regulatory genes from a gene expression data set or prognostic classifier is insufficient to adjust for the contribution of cellular proliferation in a prognostic gene list.

## Competing interests

The authors declare that they have no competing interests.

## Authors' contributions

JDM and RAK contributed equally to the design and implementation of this study. All statistical analyses were performed by JDM.

## Pre-publication history

The pre-publication history for this paper can be accessed here:



## Supplementary Material

Additional File 1 Example of the scoring procedure for two genes in a simulated gene expression data set. For each gene within the data set, a Pearson's correlation coefficient was computed for that gene vs. every other gene in the data set. In addition, a p-value for the univariate hazard ratio was also calculated for each gene. A scatter plot was constructed to compare the log of the p-value for the univariate hazard ratio for all genes versus their correlation to the individual gene being scored. Each graph shown includes only those genes that are positively correlated with the gene being scored. Each point on the graph represents the data for one of these positively correlated genes. The values for the covariance (cov) and the Pearson's correlation coefficient (corr), both computed using the data shown on the graphs, as well as the composite score are shown on the figures. The composite score was computed as follows:
Score = AbsoluteValue(Cov) * Corr
This approach was repeated for every gene in the data set. A. Scatterplot for a gene that is highly correlated with a set of genes whose expression is also associated with outcome. This is the top-scoring gene in the simulated data set. Note that many genes with a high correlation with the gene of interest also have a small significant hazards ratio p-value. B. Scatterplot for a gene receiving a low score that is not correlated with genes associated with the outcome. Click here for file

Additional File 2 Supplementary tables. Click here for file

Additional File 3 The impact of adjusting for a specific gene is determined by that gene's correlation to the cell cycle principal component variable. Scatter plot showing the impact of individually adjusting for each of the 24,495 probes in the NKI2 data set on the performance of the "70-gene" predictor. The graph is a plot of the negative log of the p-value for a univariate HR comparing good versus poor prognosis tumors after globally adjusting the gene expression data for an individual probe versus the correlation of that probe to the cell cycle PC variable. Each point on the graph shows the impact of adjusting for a single probe on the performance of the classifier. The p-value shown on the y-axis is for the univariate HR computed after the data were globally adjusted for a given gene. The graph demonstrates that adjustment for genes that are highly correlated with the cell cycle PC greatly attenuate the prognostic power of this predictor. In contrast, adjusting for genes that are not correlated with the PC, including those that comprise the predictor, has little impact on its performance. Click here for file
