# Supplementary figures and images for: Cell cycle correlated genes dictate the prognostic power of breast cancer gene lists
Source: BMC Med Genomics. 2008 Apr 25;1:11. doi: 10.1186/1755-8794-1-11 (PMC2396170; doi:10.1186/1755-8794-1-11)

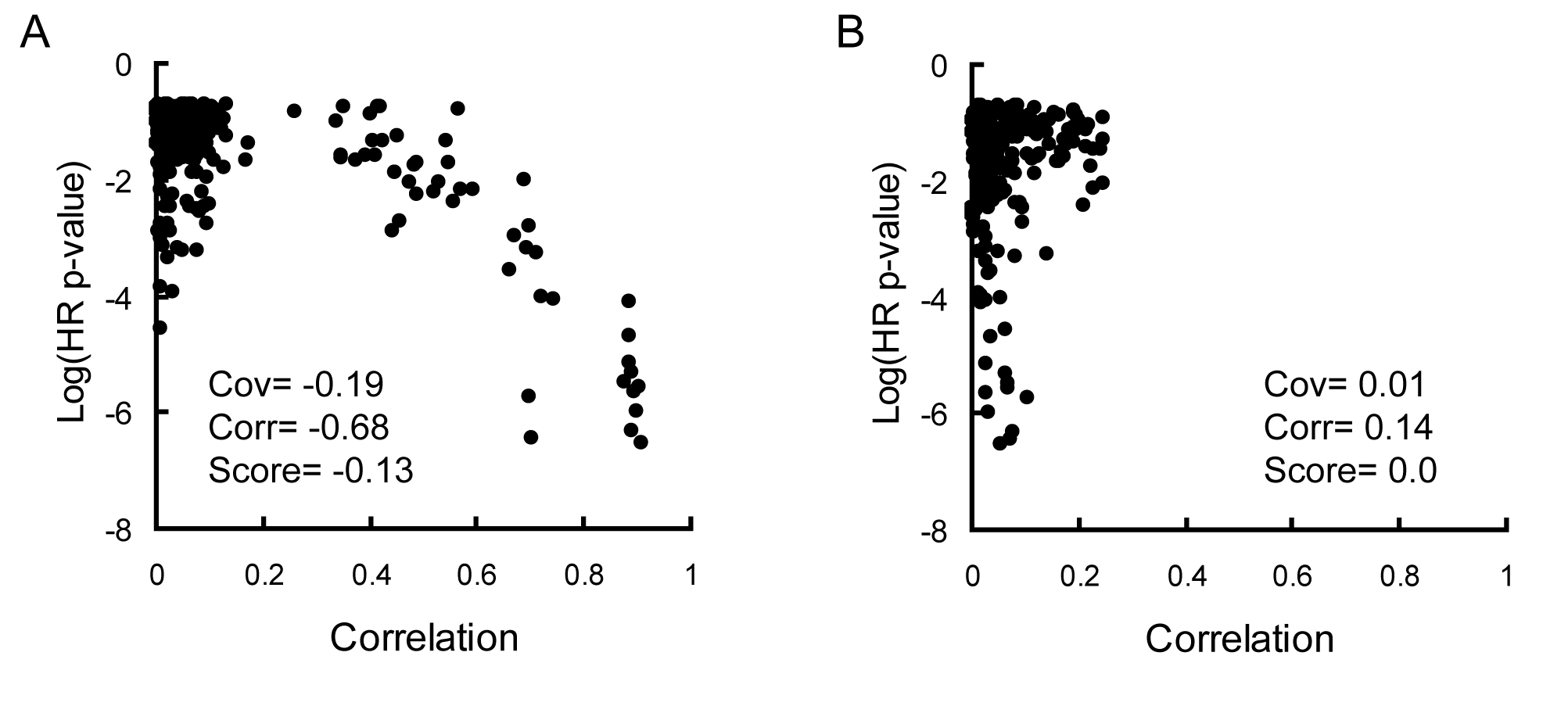

Supplement: Additional File 1 — Example of the scoring procedure for two genes in a simulated gene expression data set. For each gene within the data set, a Pearson's correlation coefficient was computed for that gene vs. every other gene in the data set. In addition, a p-value for the univariate hazard ratio was also calculated for each gene. A scatter plot was constructed to compare the log of the p-value for the univariate hazard ratio for all genes versus their correlation to the individual gene being scored. Each graph shown includes only those genes that are positively correlated with the gene being scored. Each point on the graph represents the data for one of these positively correlated genes. The values for the covariance (cov) and the Pearson's correlation coefficient (corr), both computed using the data shown on the graphs, as well as the composite score are shown on the figures. The composite score was computed as follows: Score = AbsoluteValue(Cov) * Corr This approach was repeated for every gene in the data set. A. Scatterplot for a gene that is highly correlated with a set of genes whose expression is also associated with outcome. This is the top-scoring gene in the simulated data set. Note that many genes with a high correlation with the gene of interest also have a small significant hazards ratio p-value. B. Scatterplot for a gene receiving a low score that is not correlated with genes associated with the outcome. [file 1755-8794-1-11-S1.tiff]

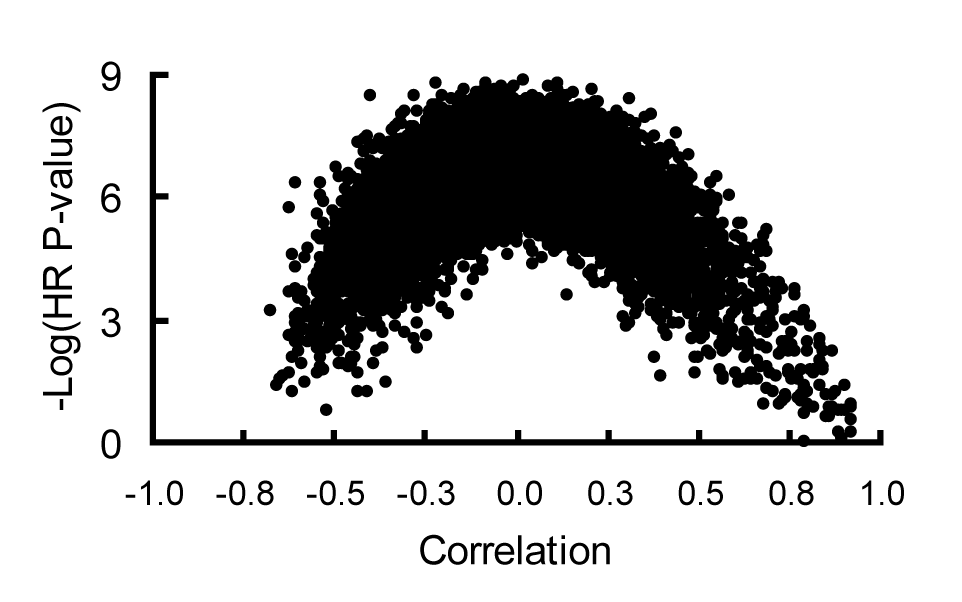

Supplement: Additional File 3 — The impact of adjusting for a specific gene is determined by that gene's correlation to the cell cycle principal component variable. Scatter plot showing the impact of individually adjusting for each of the 24,495 probes in the NKI2 data set on the performance of the "70-gene" predictor. The graph is a plot of the negative log of the p-value for a univariate HR comparing good versus poor prognosis tumors after globally adjusting the gene expression data for an individual probe versus the correlation of that probe to the cell cycle PC variable. Each point on the graph shows the impact of adjusting for a single probe on the performance of the classifier. The p-value shown on the y-axis is for the univariate HR computed after the data were globally adjusted for a given gene. The graph demonstrates that adjustment for genes that are highly correlated with the cell cycle PC greatly attenuate the prognostic power of this predictor. In contrast, adjusting for genes that are not correlated with the PC, including those that comprise the predictor, has little impact on its performance. [file 1755-8794-1-11-S3.tiff]
