# Supplementary material for: Cell cycle correlated genes dictate the prognostic power of breast cancer gene lists
Source: BMC Med Genomics. 2008 Apr 25;1:11. doi: 10.1186/1755-8794-1-11 (PMC2396170; doi:10.1186/1755-8794-1-11)
Supplement: Additional File 2 — Supplementary tables. [file 1755-8794-1-11-S2.doc]

**Supplemental Table 1.** Summary table of the number of probes examined and the number of subjects used in training sets for each published gene list analyzed.

|  |  |  | **Subjects in training sets (n)^1^** | | |
| --- | --- | --- | --- | --- | --- |
| **Data Set** | **Gene list (Ref)** | **Probes examined (n)** | **Good prognosis** | **Poor prognosis** | **Random** |
| **NKI2** |  |  |  |  |  |
|  | 70 Gene (1) | 70 | 30 | 0 | 0 |
|  | Wang 76-gene (ER+) (2) | 77 | 0 | 0 | 80 |
|  | Wang 76-gene (ER-) (2) | 21 | 0 | 0 | 35 |
|  | Wound Signature (3) | 442 | 0 | 0 | 0 |
|  | Sotiriou Grade (4) | 113 | 0 | 0 | 0 |
|  | Naderi (5) | 82 | 30 | 30 | 0 |
|  | 21 Gene Recurrence (6) | 16 | 0 | 0 | 0 |
|  |  |  |  |  |  |
| **Wang** |  |  |  |  |  |
|  | 70 Gene | 90 | 40 | 0 | 0 |
|  | Wang 76-gene (ER+) | 60 | 0 | 0 | 80 |
|  | Wang 76-gene (ER-) | 16 | 0 | 0 | 35 |
|  | Sotiriou Grade | 128 | 0 | 0 | 0 |
|  | Naderi | 102 | 40 | 30 | 0 |
|  | 21 Gene Recurrence | 39 | 0 | 0 | 0 |
|  |  |  |  |  |  |
| **KJX64/KJ125** |  |  |  |  |  |
|  | 70 Gene | 90 | 40 | 0 | 0 |
|  | Wang 76-gene (ER+) | 60 | 0 | 0 | 60 |
|  | Wang 76-gene (ER-) | 16 | 0 | 0 | 15 |
|  | Sotiriou Grade | 128 | 0 | 0 | 0 |
|  | Naderi | 102 | 40 | 20 | 0 |
|  | 21 Gene Recurrence | 39 | 0 | 0 | 0 |

Footnotes:

1. For predictive gene lists that required training sets, the numbers in these columns represent the number of subjects used that had a “good” prognosis (did not develop metastases within 5 years after diagnosis), a “poor” prognosis (had a metastatic recurrence within 5 years) or a set of randomly selected of “good” and “poor” prognosis subjects. Subjects used in training sets were not reincorporated into subsequent analyses examining the performance of the predictor.

**Supplemental Table 2:** The 25 top-ranked genes identified in the first 3 iterations of a method to identify predictive clusters of correlated genes in the NKI2 data set^1^.

| **Correlation to PC** | **Symbol** | **Unigene cluster ID** | **Name** | **Biological processes/functions** |
| --- | --- | --- | --- | --- |
| **First iteration** | | | | |
| 0.92 | UBE2C | Hs.93002 | Ubiquitin-conjugating enzyme E2C | cell cycle; cyclin catabolism; mitosis |
| 0.88 | BIRC5 | Hs.514527 | Baculoviral IAP repeat-containing 5 (survivin) | G2/M transition of mitotic cell cycle; anti-apoptosis |
| 0.87 | PKMYT1 | Hs.77783 | Protein kinase, membrane associated tyrosine/threonine 1 | cell cycle; regulation of mitosis |
| 0.93 | CDCA3 | Hs.524216 | Cell division cycle associated 3 |  |
| 0.92 | CCNB2 | Hs.194698 | Cyclin B2 | cytokinesis; mitosis; regulation of cell cycle |
| 0.90 | AURKA | Hs.250822 | Aurora kinase A | cell cycle; mitosis |
| 0.92 | DKFZp762E1312 | Hs.532968 | Hypothetical protein DKFZp762E1312 |  |
| 0.91 | BRRN1 | Hs.308045 | Barren homolog 1 (Drosophila) | mitotic cell cycle; mitotic chromosome condensation; cytokinesis |
| 0.87 | ESPL1 | Hs.153479 | Extra spindle poles like 1 (S. cerevisiae) | apoptosis; chromosome segregation |
| 0.85 | RRM2 | Hs.226390 | Ribonucleotide reductase M2 polypeptide | DNA replication |
| 0.88 | C15orf42 | Hs.591363 | ZW10 interactor | kinetochore formation; spindle checkpoint activity |
| 0.90 | KIF2C | Hs.69360 | Kinesin family member 2C | ATP binding; cell proliferation; centromeric DNA binding |
| 0.80 | TROAP | Hs.524399 | Trophinin associated protein (tastin) | cell adhesion |
| 0.86 | PRC1 | Hs.567385 | Protein regulator of cytokinesis 1 | cytokinesis; mitotic spindle elongation |
| 0.89 | KIF20A | Hs.73625 | Kinesin family member 20A | ATP binding; microtubule motor activity |
| 0.79 | TK1 | Hs.515122 | Thymidine kinase 1, soluble | DNA metabolism |
| 0.77 | UBE2S | Hs.396393 | Ubiquitin-conjugating enzyme E2S | ubiquitin cycle |
| 0.90 | CENPA | Hs.1594 | Centromere protein A | chromosome organization and biogenesis |
| 0.85 | FAM64A | Hs.592116 | Family with sequence similarity 64, member A |  |
| 0.83 | NUSAP1 | Hs.615092 | Nucleolar and spindle associated protein 1 | cytokinesis after mitosis; mitotic chromosome condensation |
| 0.86 | CDC45L | Hs.474217 | CDC45 cell division cycle 45-like (S. cerevisiae) | DNA replication; regulation of cell cycle |
| 0.86 | KIFC1 | Hs.436912 | Kinesin family member C1 | mitotic sister chromatid segregation |
| 0.79 | KPNA2 | Hs.594238 | Karyopherin alpha 2 (RAG cohort 1, importin alpha 1) | M phase specific microtubule process; NLS-bearing substrate-nucleus import |
| 0.80 | TRIP13 | Hs.436187 | Thyroid hormone receptor interactor 13 | ATP binding; nucleoside-triphosphatase activity |
| 0.73 | SPAG5 | Hs.514033 | Sperm associated antigen 5 | cell cycle; cytokinesis; mitosis |
| **Second iteration** | | | | |
| 0.64 | EEF1A1 | Hs.586423 | Eukaryotic translation elongation factor 1 alpha 1 | GTP binding; regulation of translation |
| 0.75 | NDUFA13 | Hs.534453 | NADH dehydrogenase (ubiquinone) 1 alpha subcomplex, 13 | NADH dehydrogenase activity; apoptosis; negative regulation of protein biosynthesis |
| 0.77 | RPL27A | Hs.523463 | Ribosomal protein L27a | RNA binding; protein biosynthesis |
| 0.80 | RPS6 | Hs.408073 | Ribosomal protein S6 | protein biosynthesis |
| 0.71 | RPS20 | Hs.8102 | Ribosomal protein S20 | protein biosynthesis |
| 0.66 | RPL26 | Hs.569777 | Ribosomal protein L26 |  |
| 0.67 | TPT1 | Hs.374596 | Tumor protein, translationally-controlled 1 |  |
| 0.81 | RPL11 | Hs.388664 | Ribosomal protein L11 | RNA binding; protein biosynthesis |
| 0.73 | RPSA | Hs.356262 | RPSA | cell adhesion; regulation of translation |
| 0.77 | RPS27A | Hs.546292 | Ribosomal protein S27a | Protein biosynthesis |
| 0.51 | RPL14 | Hs.446522 | Ribosomal protein L14 | RNA binding; protein biosynthesis |
| 0.69 | RPL6 | Hs.546283 | Ribosomal protein L6 | Protein biosynthesis; Regulation of transcription |
| 0.62 | RPS12 | Hs.546289 | Ribosomal protein S12 | RNA binding; protein biosynthesis |
| 0.75 | RPL5 | Hs.532359 | Ribosomal protein L5 | Protein biosynthesis |
| 0.58 | RPS8 | Hs.512675 | Ribosomal protein S8 | structural constituent of ribosome |
| 0.71 | RPS9 | Hs.546288 | Ribosomal protein S9 | Protein biosynthesis |
| 0.55 | GGN | Hs.447948 | Gametogenetin | gametogenesis; protein binding; protein localization |
| 0.47 | PCID1 | Hs.502244 | PCI domain containing 1 (herpesvirus entry mediator) |  |
| 0.60 | RPS16 | Hs.397609 | Ribosomal protein S16 | protein biosynthesis |
| 0.67 | RPS24 | Hs.356794 | Ribosomal protein S24 | protein biosynthesis |
| 0.59 | RPL13A | Hs.546356 | Ribosomal protein L13a | protein biosynthesis |
| 0.72 | RPS4X | Hs.446628 | Ribosomal protein S4, X-linked | protein biosynthesis |
| 0.75 | RPS25 | Hs.512676 | Ribosomal protein S25 | ribonucleoprotein complex |
| 0.68 | RPS14 | Hs.381126 | Ribosomal protein S14 | protein biosynthesis |
| 0.71 | RPS3A | Hs.356572 | Ribosomal protein S3A | protein biosynthesis |
| **Third iteration** | | | | |
| 0.65 | CXorf1 | Hs.106688 | Chromosome X open reading frame 1 |  |
| 0.41 | NKX3-1 | Hs.55999 | NK3 transcription factor related, locus 1 (Drosophila) | development; transcription factor activity |
| 0.68 | HADHSC | Hs.438289 | L-3-hydroxyacyl-Coenzyme A dehydrogenase, short chain | lipid metabolism |
| 0.56 | CSMD1 | Hs.571466 | CUB and Sushi multiple domains 1 | integral to membrane |
| 0.80 | TBX10 | Hs.454480 | T-box 10 | RNA polymerase II transcription factor activity; morphogenesis; transcription factor activity |
| 0.77 | ABL1 | Hs.431048 | V-abl Abelson murine leukemia viral oncogene homolog 1 | DNA damage response; S-phase-specific transcription; mismatch repair |
| 0.50 | ARF1 | Hs.286221 | ADP-ribosylation factor 1 | small GTPase mediated signal transduction |
| 0.45 | CRYGA | Hs.122566 | Crystallin, gamma A | visual perception |
| 0.69 | LOC149837 | Hs.123609 | Hypothetical protein LOC149837 |  |
| 0.24 | PCDHB14 | Hs.130757 | Protocadherin beta 14 | cell adhesion |
| 0.39 | AKAP4 | Hs.97633 | A kinase (PRKA) anchor protein 4 | cell motility; signal transduction; sperm motility |
| 0.57 | WNK2 | Hs.522291 | WNK lysine deficient protein kinase 2 | protein amino acid phosphorylation; protein kinase cascade |
| 0.50 | ST18 | Hs.147170 | Suppression of tumorigenicity 18 (breast carcinoma) (zinc finger protein) | transcription factor activity |
| 0.19 | CCDC64 | Hs.369763 | Coiled-coil domain containing 64 |  |
| 0.18 | DCAMKL2 | Hs.591683 | Doublecortin and CaM kinase-like 2 | intracellular signaling cascade |
| 0.17 | OPN3 | Hs.409081 | Opsin 3 (encephalopsin, panopsin) | Phototransduction |
| 0.25 | SOCS7 | Hs.632261 | Suppressor of cytokine signaling 7 | intracellular signaling cascade; regulation of cell growth |
| 0.30 | PSD2 | Hs.21963 | Pleckstrin and Sec7 domain containing 2 |  |
| 0.03 | HRH1 | Hs.1570 | Histamine receptor H1 | G-protein signaling, coupled to IP3 second messenger |
| 0.10 | CYP11A1 | Hs.303980 | Cytochrome P450, family 11, subfamily A, polypeptide 1 | C21-steroid hormone biosynthesis; androgen biosynthesis |
| 0.02 | TM7SF2 | Hs.31130 | Transmembrane 7 superfamily member 2 | cholesterol biosynthesis |
| 0.18 | C1orf43 | Hs.287471 | Chromosome 1 open reading frame 43 | oxidoreductase activity |
| 0.03 | LOC643797 | Hs.603731 | Hypothetical protein LOC643797 |  |
| 0.31 | GP5 | Hs.73734 | Glycoprotein V (platelet) | blood coagulation; cell adhesion |
| 0.01 | EXOC3 | Hs.481464 | Exocyst complex component 3 | Exocytosis |

Footnotes:

1. The probes are listed in ascending order by their rank. The correlation value represents the Pearson’s correlation coefficient between the expression for that gene and the principal component representing the top 10 probes in each correlated gene cluster. Blank entries in the table are due to the lack of information for a specific probe. Gene ontologies were derived from the Source database (source.stanford.edu) and Affymetrix/Netaffx (http://www.affymetrix.com/analysis/index.affx).

**Supplemental Table 3:** The 25 top-ranked genes identified in the first 2 iterations of a method to identify predictive clusters of correlated genes in the KJX64/KJ125 data sets^1^.

| **Correlation to PC** | **Symbol** | **Unigene cluster ID** | **Name** | **Biological processes/functions** |
| --- | --- | --- | --- | --- |
| **First iteration** | | | | |
| 0.90 | BUB1B | Hs.631699 | BUB1 budding uninhibited by benzimidazoles 1 homolog beta (yeast) | cell cycle; cytokinesis; mitotic checkpoint |
| 0.83 | HMMR | Hs.72550 | Hyaluronan-mediated motility receptor (RHAMM) | cell motility; hyaluronic acid binding |
| 0.85 | HMMR | Hs.72550 | Hyaluronan-mediated motility receptor (RHAMM) | cell motility; hyaluronic acid binding |
| 0.87 | KIF15 | Hs.307529 | Kinesin family member 15 | microtubule-based movement; mitosis; cell proliferation |
| 0.91 | KIF20A | Hs.73625 | Kinesin family member 20A | microtubule motor activity; protein transport |
| 0.84 | KIAA1794 | Hs.513126 | KIAA1794 |  |
| 0.85 | KIAA1794 | Hs.513126 | KIAA1794 |  |
| 0.86 | GTSE1 | Hs.386189 | G-2 and S-phase expressed 1 | DNA damage response; G2 phase of mitotic cell cycle |
| 0.86 | DKFZp762E1312 | Hs.532968 | Hypothetical protein DKFZp762E1312 |  |
| 0.88 | KIFC1 | Hs.436912 | Kinesin family member C1 | microtubule motor activity; mitotic sister chromatid segregation |
| 0.82 | LOC146909 | Hs.135094 | Hypothetical protein LOC146909 |  |
| 0.76 | NUSAP1 | Hs.615092 | Nucleolar and spindle associated protein 1 | cytokinesis after mitosis; mitotic chromosome condensation |
| 0.69 | BIRC5 | Hs.514527 | Baculoviral IAP repeat-containing 5 (survivin) | G2/M transition of mitotic cell cycle; anti-apoptosis |
| 0.76 | FOXM1 | Hs.239 | Forkhead box M1 | transcription factor activity; regulation of transcription |
| 0.72 | CDC25A | Hs.437705 | Cell division cycle 25A | cytokinesis; mitosis; regulation of cyclin dependent protein kinase activity |
| 0.77 | BLM | Hs.169348 | Bloom syndrome | ATP-dependent DNA helicase activity |
| 0.82 | STIL | Hs.525198 | SCL/TAL1 interrupting locus | cell proliferation |
| 0.68 | PSRC1 | Hs.405925 | proline/serine-rich coiled-coil 1 | cell cycle; cell division |
| 0.85 | KIF2C | Hs.69360 | Kinesin family member 2C | centromeric DNA binding; microtubule motor activity; mitosis |
| 0.86 | CEP55 | Hs.14559 | Centrosomal protein 55kDa | cell cycle; mitosis cell; division |
| 0.66 | NEIL3 | Hs.405467 | Nei endonuclease VIII-like 3 (E. coli) | DNA repair |
| 0.81 | PRC1 | Hs.567385 | Protein regulator of cytokinesis 1 | cytokinesis; mitotic spindle elongation |
| 0.72 | H2AFX | Hs.477879 | H2A histone family, member X | chromosome organization and biogenesis; nucleosome assembly |
| 0.58 | FANCG | Hs.591084 | Fanconi anemia, complementation group G | DNA repair; cell cycle checkpoint |
| 0.83 | TTK | Hs.169840 | TTK protein kinase | mitotic spindle checkpoint; positive regulation of cell proliferation |
| **Second iteration** | | | | |
| 0.88 | HLA-DRB1 | Hs.534322 | Major histocompatibility complex, class II, DR beta 1 | MHC class II receptor activity; immune response |
| 0.87 | INPP5D | Hs.262886 | Inositol polyphosphate-5-phosphatase, 145kDa | intracellular signaling cascade |
| 0.73 | NCF1 | Hs.520943 | Neutrophil cytosolic factor 1, (chronic granulomatous disease, autosomal 1) | protein biosynthesis; translation initiation factor activity |
| 0.81 | MFNG | Hs.517603 | Manic fringe homolog (Drosophila) | pattern specification |
| 0.85 | CSF2RB | Hs.592192 | Colony stimulating factor 2 receptor, beta, low-affinity (granulocyte-macrophage) | antimicrobial humoral response; receptor activity |
| 0.75 | CECR1 | Hs.170310 | Cat eye syndrome chromosome region, candidate 1 | development; purine ribonucleoside monophosphate biosynthesis |
| -0.28 | PPFIA1 | Hs.530749 | Protein tyrosine phosphatase, receptor type, f polypeptide (PTPRF), interacting protein (liprin), alpha 1 | cell-matrix adhesion; signal transduction |
| 0.66 |  | Hs.595878 | Isolate Middle91 immunoglobulin heavy chain variable region (IGVH) |  |
| 0.47 | C7orf23 | Hs.196129 | Chromosome 7 open reading frame 23 |  |
| 0.76 | HLA-DPA1 | Hs.347270 | Major histocompatibility complex, class II, DP alpha 1 | MHC class II receptor activity; antigen presentation, exogenous antigen |
| 0.68 | IGHA1 | Hs.648398 | immunoglobulin heavy constant alpha 1 | anti-apoptosis; immune response |
| 0.53 | RCBTB2 | Hs.25447 | Regulator of chromosome condensation (RCC1) and BTB (POZ) domain containing protein 2 |  |
| 0.53 | TLR8 | Hs.272410 | Toll-like receptor 8 | inflammatory response; I-kappaB kinase/NF-kappaB cascade |
| 0.58 | IGHD |  | immunoglobulin heavy constant delta | antigen processing; MHC class I immune response |
| 0.52 | MAP3K14 | Hs.404183 | Mitogen-activated protein kinase kinase kinase 14 | protein amino acid phosphorylation |
| 0.34 | IL27RA | Hs.132781 | Interleukin 27 receptor, alpha | receptor activity |
| -0.27 | LETM1 | Hs.120165 | Leucine zipper-EF-hand containing transmembrane protein 1 | development; signal transduction |
| 0.44 | GAS7 | Hs.462214 | Growth arrest-specific 7 | cell cycle arrest; cell differentiation; neurogenesis |
| 0.62 | GPSM3 | Hs.520046 | G-protein signalling modulator 3 (AGS3-like, C. elegans) | immune response; signal transduction |
| -0.09 | NAT9 | Hs.144058 | N-acetyltransferase 9 | N-acetyltransferase activity |
| 0.58 | P2RY13 | Hs.546396 | Purinergic receptor P2Y, G-protein coupled, 13 | GPCR signaling |
| 0.61 | C1S | Hs.458355 | Complement component 1, s subcomponent | complement activation, classical pathway; immune response |
| -0.20 | RSAD1 | Hs.8033 | Radical S-adenosyl methionine domain containing 1 | porphyrin biosynthesis |
| 0.54 | RGL1 | Hs.497148 | Ral guanine nucleotide dissociation stimulator-like 1 | small GTsPase mediated signal transduction |
| 0.62 | HLA-DRB1 | Hs.534322 | Major histocompatibility complex, class II, DR beta 1 | MHC class II receptor activity; immune response |

Footnotes:

1. The probes are listed in ascending order by their rank. The correlation value represents the Pearson’s correlation coefficient between the expression for that gene and the principal component representing the top 10 probes in each correlated probe cluster. Blank entries in the table are due to the lack of information for a specific probe. Gene ontologies were derived from the Source database (source.stanford.edu) and Affymetrix/Netaffx (http://www.affymetrix.com/analysis/index.affx).

**Supplemental Table 4:** The 25 top-ranked genes identified in the first 3 iterations of a method to identify predictive clusters of correlated genes in the Wang data set^1^.

| **Correlation to PC** | **Symbol** | **Unigene Cluster ID** | **Name** | **Biological processes/functions** |
| --- | --- | --- | --- | --- |
| **First iteration** | | | | |
| 0.91 | RACGAP1 | Hs.505469 | Rac GTPase activating protein 1 | GTPase activator activity; electron transport; iron ion binding |
| 0.88 | NEK2 | Hs.153704 | NIMA (never in mitosis gene a)-related kinase 2 | cytokinesis; kinase activity; regulation of mitosis |
| 0.80 | KPNA2 | Hs.594238 | Karyopherin alpha 2 (RAG cohort 1, importin alpha 1) | M phase specific microtubule process; NLS-bearing substrate-nucleus import |
| 0.84 | GINS1 | Hs.631508 | GINS complex subunit 1 (Psf1 homolog) | calcium ion binding |
| 0.82 | CSNK1G1 | Hs.81892 | Casein kinase 1, gamma 1 | Wnt receptor signaling pathway; protein kinase activity |
| 0.87 | CDC2 | Hs.334562 | Cell division cycle 2, G1 to S and G2 to M | cyclin-dependent protein kinase activity; cytokinesis; mitosis |
| 0.84 | HMMR | Hs.72550 | Hyaluronan-mediated motility receptor (RHAMM) | cell motility; hyaluronic acid binding |
| 0.86 | CCNA2 | Hs.58974 | Cyclin A2 | cytokinesis; mitotic G2 checkpoint |
| 0.80 | CCNE2 | Hs.567387 | cyclin E2 | cell cycle checkpoint; regulation of cdk activity; DNA replication initiation |
| 0.84 | CKS2 | Hs.83758 | CDC28 protein kinase regulatory subunit 2 | cell cycle; cell proliferation; cytokinesis |
| 0.87 | NUSAP1 | Hs.615092 | Nucleolar and spindle associated protein 1 | cytokinesis after mitosis; mitotic chromosome condensation |
| 0.75 | SMC4 | Hs.58992 | SMC4 structural maintenance of chromosomes 4-like 1 (yeast) | DNA replication and chromosome cycle; cytokinesis |
| 0.76 | DTL | Hs.632496 | Denticleless homolog (Drosophila) |  |
| 0.77 | RAD51AP1 |  | RAD51 associated protein 1 | DNA repair; DNA recombination |
| 0.85 | PRC1 | Hs.567385 | Protein regulator of cytokinesis 1 | cytokinesis; mitotic spindle elongation |
| 0.82 | ZWINT | Hs.591363 | ZW10 interactor | cell cycle; spindle organization and biogenesis; mitosis |
| 0.81 | AURKA | Hs.250822 | Aurora kinase A | ATP binding; cell cycle; mitosis |
| 0.60 | SMC1A | Hs.211602 | SMC1 structural maintenance of chromosomes 1-like 1 (yeast) | DNA damage response; cell cycle checkpoint; chromosome segregation; cytokinesis |
| 0.72 | RAD51 | Hs.631709 | RAD51 homolog (RecA homolog, E. coli) (S. cerevisiae) | DNA repair; DNA unwinding; mitotic recombination |
| 0.82 | AURKA |  | aurora kinase A | ATP binding; cell cycle; mitosis |
| 0.74 | KIAA1794 | Hs.513126 | KIAA1794 |  |
| 0.81 | MAD2L1 | Hs.591697 | MAD2 mitotic arrest deficient-like 1 (yeast) | cell cycle; cytokinesis; mitotic checkpoint |
| 0.70 | ECT2 | Hs.518299 | Epithelial cell transforming sequence 2 oncogene | intracellular signaling cascade; positive regulation of I-kappaB kinase/NF-kappaB cascade |
| 0.77 | KIF11 | Hs.8878 | Kinesin family member 11 | cell cycle; cytokinesis; microtubule motor activity |
| 0.84 | CCNB2 | Hs.194698 | Cyclin B2 | cytokinesis; mitosis; regulation of cell cycle |
| **Second iteration** | | | | |
| 0.91 | COL5A1 | Hs.210283 | Collagen, type V, alpha 1 | cell adhesion; extracellular matrix structural constituent |
| 0.77 | EDNRA | Hs.183713 | Endothelin receptor type A | adenylate cyclase activation; cell proliferation; phospholipase C activation |
| 0.88 | COL10A1 | Hs.520339 | collagen, type X, alpha 1(Schmid metaphyseal chondrodysplasia) | skeletal development; phosphate transport |
| 0.72 | COL5A3 | Hs.235368 | Collagen, type V, alpha 3 | cell adhesion; extracellular matrix structural constituent |
| 0.62 | LAMC1 | Hs.497039 | Laminin, gamma 1 (formerly LAMB2) | cell adhesion; positive regulation of epithelial cell proliferation |
| 0.86 | COL11A1 | Hs.523446 | Collagen, type XI, alpha 1 | cartilage condensation; cell-cell adhesion; extracellular matrix organization |
| 0.80 | NUAK1 | Hs.524692 | NUAK family, SNF1-like kinase, 1 | protein amino acid phosphorylation |
| 0.81 | TGFB1I1 | Hs.513530 | Transforming growth factor beta 1 induced transcript 1 | androgen receptor binding; positive regulation of transcription |
| 0.86 | COL11A1 | Hs.523446 | Collagen, type XI, alpha 1 | cartilage condensation; cell-cell adhesion; extracellular matrix organization |
| 0.80 | LOXL2 | Hs.626637 | Lysyl oxidase-like 2 | aging; cell adhesion; copper ion binding |
| 0.82 | CSPG2 | Hs.443681 | Chondroitin sulfate proteoglycan 2 (versican) | cell adhesion; development; cell recognition |
| 0.80 | NID2 | Hs.369840 | Nidogen 2 (osteonidogen) | basement membrane; calcium ion binding; cell-matrix adhesion |
| 0.74 | SRPX2 | Hs.306339 | Sushi-repeat-containing protein, X-linked 2 |  |
| 0.67 | MICAL2 | Hs.501928 | Microtubule associated monoxygenase, calponin and LIM domain containing 2 | cytoskeleton; electron transport; metabolism |
| 0.85 | INHBA | Hs.583348 | Inhibin, beta A (activin A, activin AB alpha polypeptide) | activin inhibitor activity; cell cycle arrest; cell differentiation; growth; TGF-B receptor binding |
| 0.75 | SULF1 | Hs.409602 | Sulfatase 1 | apoptosis; metabolism |
| 0.72 | SULF1 | Hs.409602 | Sulfatase 1 | apoptosis; metabolism |
| 0.72 | LAMA4 | Hs.213861 | Laminin, alpha 4 | blood vessel development; metabolism; regulation of cell adhesion; regulation of cell migration |
| 0.78 | DACT1 | Hs.48950 | Dapper, antagonist of beta-catenin, homolog 1 (Xenopus laevis) | development; Wnt receptor signaling pathway |
| 0.64 | HSD17B6 | Hs.524513 | Hydroxysteroid (17-beta) dehydrogenase 6 | androgen biosynthesis |
| 0.49 | P4HA2 | Hs.519568 | Procollagen-proline, 2-oxoglutarate 4-dioxygenase (proline 4-hydroxylase), alpha polypeptide II | binding; electron transporter activity |
| 0.72 | MMP11 | Hs.143751 | Matrix metallopeptidase 11 (stromelysin 3) | calcium ion binding; collagen catabolism |
| 0.48 | CMKOR1 | Hs.471751 | Chemokine orphan receptor 1 | G-protein coupled receptor activity |
| 0.20 | DKFZp667B1718 | Hs.531457 | MRNA; cDNA DKFZp667B1718 (from clone DKFZp667B1718) |  |
| 0.45 | ARHGAP1 | Hs.138860 | Rho GTPase activating protein 1 | Rho GTPase activator activity; cytoskeleton organization and biogenesis |
| **Third iteration** | | | | |
| 0.55 | PRKCBP1 |  | protein kinase C binding protein 1 | regulation of transcription, DNA-dependent |
| -0.57 | CYP2D6 | Hs.648256 | Cytochrome P450, family 2, subfamily D, polypeptide 6 | monooxygenase activity; iron ion binding |
| 0.50 | ZNF652 | Hs.463375 | Zinc finger protein 652 | nucleic acid binding; zinc ion binding |
| 0.55 | BNIP1 | Hs.145726 | BCL2/adenovirus E1B 19kDa interacting protein 1 | anti-apoptosis |
| 0.34 | DKFZp667B1718 | Hs.531457 | MRNA; cDNA DKFZp667B1718 (from clone DKFZp667B1718) |  |
| 0.64 | NOL3 | Hs.642670 | Nucleolar protein 3 (apoptosis repressor with CARD domain) |  |
| 0.39 | ZCCHC14 | Hs.156231 | Zinc finger, CCHC domain containing 14 | nucleic acid binding |
| -0.52 | CD40 | Hs.472860 | CD40 molecule, TNF receptor superfamily member 5 | protein biosynthesis; apoptosis; inflammatory response |
| 0.59 | NOL3 | Hs.642670 | Nucleolar protein 3 (apoptosis repressor with CARD domain) | mRNA processing; anti-apoptosis |
| 0.62 | EEF1A2 | Hs.433839 | Eukaryotic translation elongation factor 1 alpha 2 | protein biosynthesis; translational elongation |
| -0.29 | HLA-DRB1 | Hs.534322 | Major histocompatibility complex, class II, DR beta 1 | MHC class II receptor activity; immune response |
| -0.51 | CD8B | Hs.405667 | CD8b molecule | MHC class I protein binding; T cell activation |
| -0.46 | NFKBIE | Hs.458276 | Nuclear factor of kappa light polypeptide gene enhancer in B-cells inhibitor, epsilon | cytoplasmic sequestering of transcription factor |
| -0.45 | IGSF6 | Hs.530902 | Immunoglobulin superfamily, member 6 | immune response |
| 0.29 | GTF2H3 | Hs.355348 | General transcription factor IIH, polypeptide 3, 34kDa | damaged DNA binding; nucleotide-excision repair |
| -0.48 | MGC2463 | Hs.521075 | Hypothetical protein LOC79037 |  |
| 0.30 | RSF1 | Hs.420229 | Remodeling and spacing factor 1 | chromatin modification; histone binding; negative regulation of transcription; transcriptional activator |
| 0.40 | STX16 | Hs.307913 | Syntaxin 16 | intra-Golgi transport |
| -0.41 | MRC1 | Hs.75182 | Mannose receptor, C type 1 | pinocytosis |
| 0.19 | CLASP1 | Hs.469840 | Cytoplasmic linker associated protein 1 | mitotic chromosome condensation; negative regulation of microtubule depolymerization |
| -0.46 | LST1 | Hs.436066 | Leukocyte specific transcript 1 | cellular morphogenesis; immune response; negative regulation of lymphocyte proliferation |
| 0.30 | KIAA0513 | Hs.301658 | KIAA0513 |  |
| -0.33 | DNAJC4 | Hs.172847 | DnaJ (Hsp40) homolog, subfamily C, member 4 | protein folding |
| -0.41 | DOK2 | Hs.71215 | Docking protein 2, 56kDa | insulin receptor binding |
| -0.41 | LIMD2 | Hs.591166 | LIM domain containing 2 | zinc ion binding |

Footnotes:

1. The probes are listed in ascending order by their rank. The correlation value represents the Pearson’s correlation coefficient between the expression for that gene and the principal component representing the top 10 genes in each correlated gene cluster. Blank entries in the table are due to the lack of information for a specific probe. Gene ontologies were derived from the Source database (source.stanford.edu) and Affymetrix/Netaffx (http://www.affymetrix.com/analysis/index.affx).

**Supplemental Table 5:** The 25 top-ranked genes identified in the first 2 iterations of a method to identify predictive clusters of correlated genes in the TRANSBIG^1^ data set^2^.

| **Correlation to PC** | **Symbol** | | **Unigene Cluster ID** | **Name** | **Biological processes/functions** |
| --- | --- | --- | --- | --- | --- |
| **First iteration** | | | |  |  |
| 0.92 | | UBE2C | Hs.93002 | Ubiquitin-conjugating enzyme E2C | cell cycle; cyclin catabolism; mitosis |
| 0.90 | | KIF4A | Hs.648326 | Kinesin family member 4A | DNA binding; microtubule-based movement; spindle microtubule |
| 0.87 | | CDKN3 | Hs.84113 | Cyclin-dependent kinase inhibitor 3 (CDK2-associated dual specificity phosphatase) | cell cycle; negative regulation of cell proliferation; regulation of cdk activity |
| 0.88 | | AURKA | Hs.250822 | Aurora kinase A | ATP binding; cell cycle; mitosis |
| 0.85 | | ZWINT | Hs.591363 | ZW10 interactor | cell cycle; spindle organization and biogenesis; mitosis |
| 0.90 | | KIF2C | Hs.69360 | Kinesin family member 2C | ATP binding; cell proliferation; centromeric DNA binding |
| 0.89 | | RACGAP1 | Hs.696319 | Rac GTPase activating protein 1 | GTPase activator activity; electron transport; iron ion binding |
| 0.92 | | NUSAP1 | Hs.615092 | Nucleolar and spindle associated protein 1 | cytokinesis after mitosis; mitotic chromosome condensation |
| 0.88 | | BUB1 | Hs.469649 | BUB1 budding uninhibited by benzimidazoles 1 homolog (yeast) | cell cycle; cell proliferation; cytokinesis; mitosis |
| 0.90 | | PRC1 | Hs.567385 | Protein regulator of cytokinesis 1 | cytokinesis; mitotic spindle elongation |
| 0.89 | | CENPA | Hs.1594 | Centromere protein A | chromosome organization and biogenesis |
| 0.82 | | TOP2A | Hs.156346 | Topoisomerase (DNA) II alpha 170kDa | ATP binding; DNA topoisomerase activity |
| 0.83 | | KIF20A | Hs.73625 | Kinesin family member 20A | ATP binding; microtubule motor activity |
| 0.86 | | MELK | Hs.184339 | Maternal embryonic leucine zipper kinase | ATP binding; phosphorylation |
| 0.90 | | CCNB2 | Hs.194698 | Cyclin B2 | cytokinesis; mitosis; regulation of cell cycle |
| 0.88 | | AURKA | Hs.250822 | Aurora kinase A | ATP binding; cell cycle; mitosis |
| 0.79 | | DTL | Hs.656473 | Denticleless homolog (Drosophila) |  |
| 0.75 | | ASF1B | Hs.26516 | ASF1 anti-silencing function 1 homolog B (S. cerevisiae) | chromatin assembly; histone binding |
| 0.81 | | GINS1 | Hs.658464 | GINS complex subunit 1 (Psf1 homolog) | calcium ion binding |
| 0.84 | | RRM2 | Hs.226390 | Ribonucleotide reductase M2 polypeptide | DNA replication; deoxyribonucleoside diphosphate metabolism |
| 0.74 | | SPAG5 | Hs.514033 | Sperm associated antigen 5 | cell cycle; cytokinesis; mitosis |
| 0.86 | | CCNB1 | Hs.23960 | Cyclin B1 | G2/M transition of mitotic cell cycle; cytokinesis |
| 0.82 | | NEK2 | Hs.153704 | NIMA (never in mitosis gene a)-related kinase 2 | centrosome; cytokinesis; magnesium ion binding |
| 0.77 | | BRRN1 | Hs.308045 | Barren homolog 1 (Drosophila) | Mitotic cell cycleIMitotic chromosome condensationICytokinesis |
| 0.84 | | CDCA8 | Hs.524571 | Cell division cycle associated 8 | cytokinesis |
| **Second iteration** | | |  |  |  |
| 0.70 | | FN1 | Hs.203717 | Fibronectin 1 | cell adhesion; cell migration; Wounding |
| -0.24 | | ARL6IP5 | Hs.92384 | ADP-ribosylation-like factor 6 interacting protein 5 | glutamate transport |
| -0.24 | | GLRX | Hs.28988 | Glutaredoxin (thioltransferase) | cell redox homeostasis |
| 0.19 | | ERO1L | Hs.592304 | ERO1-like (S. cerevisiae) | electron transport; oxidoreductase activity |
| 0.72 | | SERPINE1 | Hs.414795 | Serpin peptidase inhibitor, clade E (nexin, plasminogen activator inhibitor type 1), member 1 | blood coagulation; plasminogen activator activity |
| 0.67 | | TNFAIP6 | Hs.437322 | Tumor necrosis factor, alpha-induced protein 6 | cell adhesion; cell-cell signaling; inflammatory response |
| 0.76 | | GREM1 | Hs.40098 | Gremlin 1, cysteine knot superfamily, homolog (Xenopus laevis) | cytokine activity; development; neurogenesis |
| 0.18 | | FAM120A | Hs.76666 | family with sequence similarity 120A |  |
| 0.35 | | MARCH6 | Hs.432862 | Membrane-associated ring finger (C3HC4) 6 | protein ubiquitination |
| 0.43 | | FGFR1 | Hs.264887 | Fibroblast growth factor receptor 1 (fms-related tyrosine kinase 2, Pfeiffer syndrome) | MAPKKK cascade; cell growth |
| 0.43 | | XYLT1 | Hs.22907 | xylosyltransferase I | glycosaminoglycan biosynthetic process |
| 0.64 | | TNFAIP6 | Hs.437322 | Tumor necrosis factor, alpha-induced protein 6 | cell adhesion; cell-cell signaling; inflammatory response |
| 0.20 | | PKMYT1 | Hs.77783 | Protein kinase, membrane associated tyrosine/threonine 1 | cell cycle; regulation of mitosis |
| 0.14 | | PAEP | Hs.532325 | Progestagen-associated endometrial protein (placental protein 14, pregnancy-associated endometrial alpha-2-globulin, alpha uterine protein) | development; transport |
| 0.39 | | CMKOR1 | Hs.471751 | Chemokine orphan receptor 1 | G-protein coupled receptor activity |
| 0.13 | | SFRS15 | Hs.17255 | Splicing factor, arginine/serine-rich 15 | RNA binding |
| 0.11 | | GNRH2 | Hs.129715 | Gonadotropin-releasing hormone 2 | development; hormone activity; signal transduction |
| 0.66 | | MMP11 | Hs.143751 | Matrix metallopeptidase 11 (stromelysin 3) | collagen catabolism; morphogenesis |
| 0.21 | | KIAA1005 | RPGRIP1L | RPGRIP1-like |  |
| 0.39 | | TWIST1 | Hs.66744 | Twist homolog 1 (acrocephalosyndactyly 3; Saethre-Chotzen syndrome) (Drosophila) | RNA polymerase II transcription factor activity; cell differentiation |
| 0.55 | | RGS4 | Hs.386726 | Regulator of G-protein signaling 4 | GTPase activator activity; calmodulin binding; signal transduction |
| -0.15 | | ACP5 | Hs.1211 | Acid phosphatase 5, tartrate resistant | acid phosphatase activity |
| -0.13 | | RTN1 | Hs.368626 | Reticulon 1 | neuron differentiation; signal transducer activity |
| 0.07 | | PHF8 | Hs.133352 | PHD finger protein 8 | regulation of transcription, DNA-dependent |
| -0.18 | | HOXA2 | Hs.58116 | homeobox A2 | cell fate determination; regulation of transcription |

Footnotes:

1. The TRANSBIG (7) data set is described in supplemental table 7.
2. The probes are listed in ascending order by their rank. The correlation value represents the Pearson’s correlation coefficient between the expression for that gene and the principal component representing the top 10 genes in each correlated gene cluster. Blank entries in the table are due to the lack of information for a specific probe. Gene ontologies were derived from the Source database (source.stanford.edu) and Affymetrix/Netaffx (http://www.affymetrix.com/analysis/index.affx).

**Supplemental Table 6:** The 25 top-ranked genes identified in the first 2 iterations of a method to identify predictive clusters of correlated genes in the Stockholm^1^ data set^2^.

| **Correlation to PC** | **Symbol** | | **Unigene Cluster ID** | **Name** | **Biological processes/functions** |
| --- | --- | --- | --- | --- | --- |
| **First iteration** | | | |  |  |
| 0.93 | | TPX2 | Hs.620407 | TPX2, microtubule-associated, homolog (Xenopus laevis) | cell proliferation; mitosis; spindle |
| 0.84 | | HN1 | Hs.532803 | hematological and neurological expressed 1 |  |
| 0.93 | | RRM2 | Hs.226390 | Ribonucleotide reductase M2 polypeptide | DNA replication |
| 0.93 | | CCNB2 | Hs.194698 | Cyclin B2 | cytokinesis; mitosis; regulation of cell cycle |
| 0.86 | | KPNA2 | Hs.594238 | Karyopherin alpha 2 (RAG cohort 1, importin alpha 1) | M phase specific microtubule process; NLS-bearing substrate-nucleus import |
| 0.88 | | FOXM1 | Hs.239 | Forkhead box M1 | transcription factor activity; regulation of transcription |
| 0.91 | | PRC1 | Hs.567385 | Protein regulator of cytokinesis 1 | cytokinesis; mitotic spindle elongation |
| 0.92 | | RRM2 | Hs.226390 | Ribonucleotide reductase M2 polypeptide | DNA replication |
| 0.82 | | SHCBP1 | Hs.123253 | SHC SH2-domain binding protein 1 | protein binding |
| 0.90 | | CCNB1 | Hs.23960 | Cyclin B1 | G2/M transition of mitotic cell cycle; cytokinesis |
| 0.89 | | CDC20 | Hs.524947 | Cell division cycle 20 homolog (S. cerevisiae) | cytokinesis; mitosis; regulation of cell cycle; ubiquitin cycle |
| 0.80 | | H2AFZ | Hs.119192 | H2A histone family, member Z | nucleosome assembly; chromosome organization and biogenesis |
| 0.69 | | PGK1 | Hs.78771 | Phosphoglycerate kinase 1 | ATP binding; glycolysis |
| 0.58 | | KARS | Hs.3100 | Lysyl-tRNA synthetase | aspartate-tRNA ligase activity |
| 0.85 | | BIRC5 | Hs.514527 | Baculoviral IAP repeat-containing 5 (survivin) | G2/M transition of mitotic cell cycle; anti-apoptosis |
| 0.88 | | RACGAP1 | Hs.696319 | Rac GTPase activating protein 1 | GTPase activator activity; electron transport; iron ion binding |
| 0.81 | | H2AFZ | Hs.119192 | H2A histone family, member Z | Histone |
| 0.85 | | UBE2S | Hs.396393 | Ubiquitin-conjugating enzyme E2S | Ubiquitin cycle |
| 0.90 | | UBE2C | Hs.93002 | Ubiquitin-conjugating enzyme E2C | cell cycle; cyclin catabolism; mitosis |
| 0.75 | | DDX39 | Hs.311609 | DEAD (Asp-Glu-Ala-Asp) box polypeptide 39 | ATP-dependent RNA helicase activity; nuclear mRNA splicing, via spliceosome |
| 0.87 | | CSNK1G1 | Hs.646508 | Casein kinase 1, gamma 1 | Wnt receptor signaling pathway; protein amino acid phosphorylation |
| 0.88 | | PTTG1 | Hs.350966 | Pituitary tumor-transforming 1 | DNA replication and chromosome cycle; cytokinesis |
| 0.77 | | RNASEH2A | Hs.532851 | Ribonuclease H2, subunit A | DNA replication; RNA binding |
| 0.86 | | BUB1B | Hs.631699 | BUB1 budding uninhibited by benzimidazoles 1 homolog beta (yeast) | cell cycle; cytokinesis; mitotic checkpoint |
| 0.87 | | AURKA | Hs.250822 | Aurora kinase A | ATP binding; cell cycle; mitosis |
| **Second iteration** | | |  |  |  |
| 0.61 | | NFATC3 | Hs.632209 | Nuclear factor of activated T-cells, cytoplasmic, calcineurin-dependent 3 | inflammatory response; regulation of transcription |
| 0.51 | | VAC14 | Hs.445061 | Vac14 homolog (S. cerevisiae) | receptor activity; signal transduction |
| 0.71 | | MAP1LC3B | Hs.356061 | Microtubule-associated protein 1 light chain 3 beta | autophagy; ubiquitin cycle |
| 0.52 | | TRADD | Hs.460996 | TNFRSF1A-associated via death domain | apoptosis; regulation of I-kappaB kinase |
| 0.53 | | FAM38A | Hs.513807 | Family with sequence similarity 38, member A |  |
| 0.73 | | POLR2C | Hs.79402 | Polymerase (RNA) II (DNA directed) polypeptide C, 33kDa | RNA polymerase II, core complex |
| 0.41 | | ATP2C2 | Hs.6168 | ATPase, Ca++ transporting, type 2C, member 2 | cation transport |
| 0.59 | | SPG7 | Hs.185597 | Spastic paraplegia 7 (pure and complicated autosomal recessive) | cell adhesion; metalloendopeptidase activity |
| 0.71 | | HSPC171 | Hs.433203 | HSPC171 protein |  |
| 0.73 | | COQ9 | Hs.513632 | Coenzyme Q9 homolog (S. cerevisiae) | ubiquinone biosynthetic process |
| 0.21 | | IL1RN | Hs.81134 | Interleukin 1 receptor antagonist | inflammatory response |
| 0.14 | | SERPINE1 | Hs.414795 | Serpin peptidase inhibitor, clade E (nexin, plasminogen activator inhibitor type 1), member 1 | blood coagulation; plasminogen activator activity |
| 0.73 | | CIAPIN1 | Hs.4900 | Cytokine induced apoptosis inhibitor 1 | anti-apoptosis; methyltransferase |
| 0.61 | | DDX19B | Hs.221761 | DEAD (Asp-Glu-Ala-As) box polypeptide 19B | ATP-dependent helicase |
| -0.26 | | MAPK8 | Hs.138211 | Mitogen-activated protein kinase 8 | JNK cascade; JUN kinase activity; cell motility; response to stress |
| 0.35 | | KLF6 | Hs.4055 | Kruppel-like factor 6 | B cell differentiation; cell growth; regulation of transcription |
| 0.35 | | CIAPIN1 | Hs.4900 | Cytokine induced apoptosis inhibitor 1 | anti-apoptosis; methyltransferase |
| 0.59 | | CNOT1 | Hs.279949 | CCR4-NOT transcription complex, subunit 1 | protein binding |
| 0.65 | | AARS | Hs.315137 | Alanyl-tRNA synthetase | alanine-tRNA ligase activity; tRNA binding |
| 0.44 | | MLYCD | Hs.644610 | malonyl-CoA decarboxylase | fatty acid biosynthetic process |
| 0.62 | | ATP6V0D1 | Hs.106876 | ATPase, H+ transporting, lysosomal 38kDa, V0 subunit d1 | ATP synthesis coupled proton transport |
| -0.17 | | TTTY15 | Hs.433656 | Testis-specific transcript, Y-linked 15 |  |
| 0.23 | | NBEAL2 | Hs.437043 | Neurobeachin-like 2 | immune response; receptor linked signal transduction |
| 0.58 | | MBTPS1 | Hs.75890 | Membrane-bound transcription factor peptidase, site 1 | cholesterol metabolism; peptidase activity |
| -0.23 | | DKFZp686O1327 | Hs.42192 | Hypothetical gene supported by BC043549; BX648102 |  |

Footnotes:

1. The Stockholm (8) data set is described in supplemental table 7.
2. The probes are listed in ascending order by their rank. The correlation value represents the Pearson’s correlation coefficient between the expression for that gene and the principal component representing the top 10 genes in each correlated gene cluster. Blank entries in the table are due to the lack of information for a specific probe. Gene ontologies were derived from the Source database (source.stanford.edu) and Affymetrix/Netaffx (http://www.affymetrix.com/analysis/index.affx).

**Supplemental Table 7:** Performance of the principal component variables as prognostic classifiers^1^.

| **Data Set** | **Principal component** | **Good**^2^ | **Poor**^2^ | **HR** | **95% CI** | **p-value** |
| --- | --- | --- | --- | --- | --- | --- |
| NKI2 |  |  |  |  |  |  |
|  | PC1 | 103 | 192 | 5.8 | (2.9-11.6) | <0.0001 |
|  | PC2 | 53 | 242 | 2.1 | (1.0-4.2) | 0.04 |
|  | PC3 | 50 | 245 | 1.9 | (0.9-3.7) | 0.08 |
|  |  |  |  |  |  |  |
| Wang |  |  |  |  |  |  |
|  | PC1 | 67 | 219 | 3.1 | (1.6-5.9) | 0.0008 |
|  | PC2 | 44 | 242 | 1.7 | (0.9-3.2) | 0.12 |
|  | PC3 | 50 | 236 | 1.9 | (1.0-3.7) | 0.05 |
|  |  |  |  |  |  |  |
| KJX64/KJ125 |  |  |  |  |  |  |
|  | PC1 | 58 | 131 | 3.9 | (1.5-10.0) | 0.004 |
|  | PC2 | 42 | 147 | 2.5 | (1.0-6.5) | 0.05 |
|  |  |  |  |  |  |  |
| TRANSBIG^3^ |  |  |  |  |  |  |
|  | PC1 | 90 | 108 | 7.4 | (2.6-20.8) | 0.0002 |
|  | PC2 | 78 | 120 | 5.6 | (2.0-15.8) | 0.001 |
|  |  |  |  |  |  |  |
| Stockholm^4^ |  |  |  |  |  |  |
|  | PC1 | 61 | 98 | 5.7 | (2.0-16.0) | 0.001 |
|  | PC2 | 42 | 117 | 3.1 | (1.1-8.9) | 0.03 |

Footnotes:

1. Hazard ratios (HR), 95% confidence intervals (CI) and p-values represent the change in risk for tumors classified as having a poor prognosis versus tumors classified as having a good prognosis using a univariate proportional hazards analysis. Tumors were separately classified into good or poor prognosis groups for each principal component (PC) variable. Classification into groups was based on a tumor’s value for the PC variable using a 90% sensitivity (10% false positive) threshold for group determination.
2. Values in the Good and Poor columns indicate the number of tumors assigned to the good and poor prognosis groups, respectively.
3. The TRANSBIG data set contains gene expression data on 198 women with lymph node negative disease (7) (downloaded from <http://www.ncbi.nlm.nih.gov/geo/>, series GSE73904). This data set did not include information about the ER status of the tumors, precluding an analysis of the Wang classifier.
4. The Stockholm data set contains gene expression data on 159 women with or without adjuvant systemic treatment (8) (downloaded from <http://www.ncbi.nlm.nih.gov/geo/>, series GSE1456).

**Supplemental Table 8:** Prognostic gene lists rely on genes correlated with the first (cell cycle) principal component variable in 2 additional independent data sets^1^.

|  |  | **Intercept adjusted** | | **PC1 adjusted** | | **PC2 adjusted** | |
| --- | --- | --- | --- | --- | --- | --- | --- |
| **Data set** | **Gene list** | **Good/Poor^2^** | **HR**  **(p-value)** | **Good/Poor^2^** | **HR**  **(p-value)** | **Good/Poor^2^** | **HR**  **(p-value)** |
| **TRANSBIG** | |  |  |  |  |  |  |
|  | 70 Gene | 46 / 112 | **3.5 (0.02)** | 14 / 144 | 0.7 (0.51) | 61 / 97 | **5.5 (0.001)** |
|  | Wang 76-gene (ER+) | 67 / 17 | **4 (0.03)** | 37 / 47 | 1.9 (0.36) | 70 / 14 | **3.5 (0.05)** |
|  | Wang 76-gene (ER-) | 13 / 21 | 0.7 (0.45) | 14 / 20 | 0.6 (0.59) | 13 / 21 | 0.62 (0.45) |
|  | Sotiriou Grade | 94 / 104 | **7.9 (0.0001)** | 38 / 160 | 2 (0.20) | 94 / 104 | **7.9 (0.0001)** |
|  | Naderi | 81 / 72 | **2.9 (0.03)** | 73 / 80 | 1.2 (0.74) | 80 / 73 | **4 (0.005)** |
| **Stockholm** | |  |  |  |  |  |  |
|  | 70 Gene^3^ | 61 / 83 | **3.5 (0.003)** | 26 / 118 | 0.9 (0.74) | 62 / 82 | **3.6 (0.003)** |
|  | Sotiriou Grade | 60 / 99 | **5.4 (0.002)** | 16 / 143 | 0.9 (0.79) | 60 / 99 | **5.4 (0.002)** |
|  | Naderi | 69 / 60 | **3.8 (0.01)** | 65 / 64 | 1.2 (0.63) | 69 / 60 | **3.8 (0.01)** |

Footnotes:

1. Hazard ratios (HR) represent the change in risk for tumors classified as having a poor prognosis versus tumors classified as having a good prognosis using a univariate proportional hazards analysis. Gene expression data were independently adjusted using simple linear regression analysis for either an intercept only, the principal component representing the first correlated gene cluster identified (PC1) or the second correlated gene cluster identified (PC2) in each data set. Univariate HRs that are significantly (p<0.05) greater than 1 are shown in bold.
2. Values in the Good/Poor column indicate the number of tumors assigned to the good and poor prognosis groups, respectively. The total number of tumors per gene list will vary depending upon the number of tumors used in training sets.
3. The 70-gene classifier did not perform appropriately using a 90% sensitivity threshold. Hence, an 80% sensitivity threshold was used for patient classification.

**REFERENCES**

1. van de Vijver MJ, He YD, van't Veer LJ, et al. A gene-expression signature as a predictor of survival in breast cancer. N.Engl.J Med. 2002;*347*:1999-2009.

2. Wang Y, Klijn JG, Zhang Y, et al. Gene-expression profiles to predict distant metastasis of lymph-node-negative primary breast cancer. Lancet 2005;*365*:671-9.

3. Chang HY, Nuyten DS, Sneddon JB, et al. Robustness, scalability, and integration of a wound-response gene expression signature in predicting breast cancer survival. Proc Natl Acad Sci U.S.A 2005;*102*:3738-43.

4. Sotiriou C, Wirapati P, Loi S, et al. Gene expression profiling in breast cancer: understanding the molecular basis of histologic grade to improve prognosis. J Natl Cancer Inst. 2006;*98*:262-72.

5. Naderi A, Teschendorff AE, Barbosa-Morais NL, et al. A gene-expression signature to predict survival in breast cancer across independent data sets. Oncogene 2006;*26*:1507-16.

6. Paik S, Shak S, Tang G, et al. A multigene assay to predict recurrence of tamoxifen-treated, node-negative breast cancer. N.Engl.J Med. 2004;*351*:2817-26.

7. Desmedt C, Piette F, Loi S, et al. Strong time dependence of the 76-gene prognostic signature for node-negative breast cancer patients in the TRANSBIG multicenter independent validation series. Clin.Cancer Res. 2007;*13*:3207-14.

8. Pawitan Y, Bjohle J, Amler L, et al. Gene expression profiling spares early breast cancer patients from adjuvant therapy: derived and validated in two population-based cohorts. Breast Cancer Res. 2005;*7*:R953-R964.
